# Liver-on-a-Chip (LoC) Models: Case Studies of Academic Platforms and Commercial Products

**DOI:** 10.1021/acs.molpharmaceut.5c01122

**Published:** 2026-02-02

**Authors:** Zineb Benzait, Özlem Tomsuk, Aliakbar Ebrahimi, Hamed Ghorbanpoor, Ceren Özel, Reza Didarian, Bahar Demir Cevizlidere, Murat Kaya, Tamer Gur, Nigar Gasimzade, Fulya Büge Ergen, Ahmet Emin Topal, Shadab Dabagh, Roshan Javanifar, Nuran Abdullayeva, Onur Uysal, Ayla Eker Sarıboyacı, Fatma Doğan Güzel, Shabir Hassan, Huseyin Avci

**Affiliations:** 1 Cellular Therapy and Stem Cell Production Application and Research Center (ESTEM), 53004 Eskisehir Osmangazi University, Eskisehir 26040, Türkiye; 2 Energy Efficient Composite Materials Laboratory, Engineering Department, Karadeniz Technical University, Trabzon 61080, Türkiye; 3 Montpellier Cancer Research Institute (IRCM), University of Montpellier, Montpellier 34298, France; 4 Department of Mechanical Engineering, Middle East Technical University, Ankara 06800, Türkiye; 5 Department of Biomedical Engineering, 226850 Ankara Yıldırım Beyazıt University, Ankara 06010, Türkiye; 6 Department of Biomedical Engineering, Eskisehir Osmangazi University, Eskisehir 26040, Türkiye; 7 Division of Engineering in Medicine, Brigham and Women’s Hospital, Harvard Medical School, Cambridge, Boston, Massachusetts 02115, United States; 8 Department of Stem Cell, Institute of Health Sciences, Eskisehir Osmangazi University, Eskisehir 26040, Türkiye; 9 Department of Biology, 26567 University of Konstanz, Konstanz 78457, Germany; 10 Scientific - Research Institute of Medical Prophylaxis Named after V. Y. Akhundov, Baku AZ1065, Azerbaijan; 11 Biochemistry Department, School of Pharmacy, 52946 Bahçeşehir University, Istanbul 34353, Türkiye; 12 Institute of Applied Physics Nello Carrara, National Research Council of Italy (CNR), Sesto Fiorentino 50019, Italy; 13 Institute of Science, Polymer Science and Technology Department, Eskisehir Osmangazi University, Eskisehir 26040, Türkiye; 14 Department of Biological Sciences, Khalifa University, Abu Dhabi 127788, United Arab Emirates; 15 Center for Biotechnology, Khalifa University, Abu Dhabi 127788, United Arab Emirates; 16 Department of Metallurgical and Materials Engineering, Eskisehir Osmangazi University, Eskisehir 26040, Türkiye; 17 Translational Medicine Research and Clinical Center (TATUM), Eskisehir Osmangazi University, Eskisehir 26040, Türkiye

**Keywords:** liver-on-a-chip, organ-on-a-chip, microfluidics, liver modeling, OoC technology, microphysiological systems

## Abstract

Pharmaceutical companies place significant importance on the liver due to its crucial role in numerous biochemical processes, specifically in drug metabolism. This focus has led to significant progress in liver-on-a-chip (LoC) technology, which has proven useful not only in drug development but also in more advanced applications. As a result, elaboration and incorporation of advanced LoC models into preclinical workflows have great potential to decrease R&D expenses and reduce or even replace animal testing, while improving the safety and efficacy of new therapies. To explore this potential, the present review provides an overview of recent academic and commercial LoC models, examines their different designs and cellular compositions, and evaluates the advantages and disadvantages of their complexity. A systematic comparison of these models is then performed, along with a discussion of their current challenges and future perspectives. Ultimately, we hope this review will assist scientists and industry professionals in selecting optimal models and in contributing to future advancements in LoC technology.

## Graphical abstract

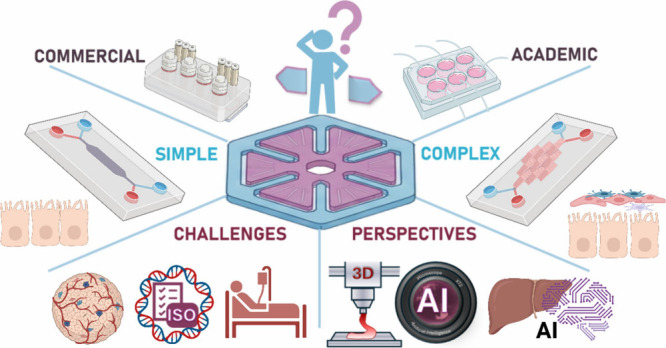

## 1 Introduction

For over 500 functions including metabolism, detoxification, storage, and synthesis of proteins, the liver is the largest and one of the most vital organs.^1^ Owing to its role in drug metabolism, the liver is an intense focus of pharmaceutical companies, as a large number of registered drug candidates fail in clinical trials due to unpredicted hepatotoxicity.^2^ Furthermore, many pharmaceuticals are withdrawn from the market because of drug-induced liver injury (DILI).^3^ Therefore, establishing comprehensive models capable of accurately mimicking human liver physiology is highly required to investigate its operational mechanisms and reactions to diverse substances.

Traditional animal testing has shown inaccuracy, raises numerous ethical concerns, and is associated with high drug development costs.^4,5^ More relevant models, such as liver-on-a-chip (LoC) systems, significantly address these issues.^6–8^ This is best emphasized by the U.S. Food and Drug Administration (FDA) Modernization Act 2.0, which permitted the registration of drugs without animal testing if other predictive models are involved, including organ-on-chips (OoC).^9,10^ Such legislative change is crucial for promoting OoC technologies, particularly LoCs, currently considered among the most advanced in vitro models.^11–13^ This recognition has consequently encouraged academics and drug manufacturers to conduct further research aimed at investing in and/or adopting these novel systems.

While there is great interest in OoC, especially LoCs, it is essential to carefully consider whether this enthusiasm is justified or overly optimiztic. Recent studies indicate that many LoC models could significantly enhance the predictive accuracy for DILI and other hepatotoxic effects and, therefore, hold a promise of revolutionizing preclinical testing.^1,6,14^ Additionally, integrating LoCs into preclinical workflows can reduce R&D costs by up to 26%^15^ and generate more than $3 billion annually.^6^ LoC technology extends beyond pharmaceutical applications; it holds a promise for disease modeling,^16^ personalized medicine,^17^ and even environmental toxicology.^18^ This broad versatility underlines the need to enhance LoC platforms. In this context, we reviewed various models recently developed in research laboratories. Notably, these academic models are different in design, ranging from simple to complicated architectures replicating hepatic lobules, and feature various cell configurations, ranging from mono- to quad-cultures. Hence, determining the advantages and disadvantages of complex ones is important for their selection in specific applications and further pursuing their possible transition to industrial manufacturing, a topic that will also be discussed within this review.

Case studies of current commercial LoC products, such as Emulate and CN-Bio, are also presented, highlighting their key features and successful implementation in various applications. Although many startups have emerged from academic laboratories through huge developments, several similarities persist in certain academic and commercial LoC models. This review systematically compares these two categories, with the aim of guiding both researchers and industry leaders toward improving the efficacy of liver research and directing future developments in LoC technology. Many current challenges must be addressed, including reproducibility, monitoring, data analysis, and validation. However, there are numerous prospects discussed at the end of the review, which emphasize that LoC technology remains holds beyond the hype, significant promise across multiple applications. In this regard, collaboration among academia, industry stakeholders, and policymakers is essential for overcoming existing obstacles while fully exploiting the potential of LoC technology.

## 2 Importance of Mimicking Liver

### 2.1 Liver Structure and Microenvironment

As the largest organ in the human body, the liver is essential for many critical physiological functions. Its complex microenvironment and physical structure are intricately organized, enabling it to perform these tasks efficiently and consistently. The liver is composed of a parenchymal mass with two major lobes, right and left (Figure 1a), traversed by an intricate system of blood networks that resemble tunnel structures known as lacunes.^19^

**Figure 1:**
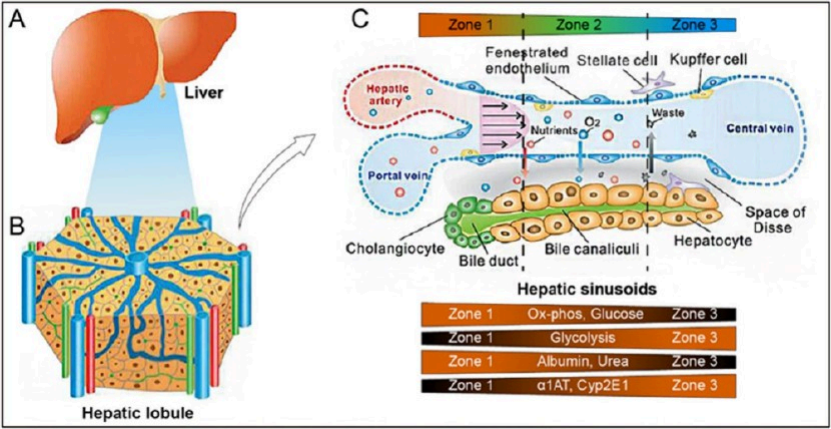
Schematic of the liver’s anatomy and components: A) the entire liver. B) The liver lobule. C) The liver sinusoid and the phenomena of zonation. Reproduced from ref.^34^ with permission from the Royal Society of Chemistry..

#### 2.1.1 Vascularization

The liver is a highly vascularized organ that functions as a significant blood reservoir, holding approximately 10–15% of the body’s total blood volume. Physiologically, it receives approximately 25% of the resting cardiac output (approximately 800–1200 mL/min).^20^ The blood supply is dual in nature: the portal vein (PV) contributes roughly 75–80% of the inflow, delivering nutrient-rich but partially oxygen-depleted blood from the splanchnic bed, while the hepatic artery (HA) delivers the remaining 20–25% of well-oxygenated blood.^20,21^ The portal venous system’s branching pattern, described by the ″1–2–20″ paradigm,^22^ feeds into the sinusoidal network which accounts for 60% of the vascular volume.^23^ These sinusoids perfuse a uniquely organized parenchyma. Specifically, it is not composed of simple cords, but of thin hepatic plates (laminae) that are typically one cell thick, a defining feature of the human liver that distinguishes it from lower vertebrates.^19,21^ This specialized design effectively maximizes the interfacial contact area of hepatocytes (HCs), the parenchymal liver cells, with the perfusing blood, ensuring direct feeding and rapid metabolic exchange. But what exactly are these sinusoids?

#### 2.1.2 Hepatic Lobule and Sinusoids

The hepatic lobule is the liver’s functional unit and has a hexagonal prism shape (Figure 1b). The human liver contains approximately10^5^ −10^6^ lobules,^24,25^ which range from 500 μm to 2.5 mm in diameter.^21,26–29^ The central vein (CV), which drains blood from the liver, is located at the center each lobule, whereas the portal triads (containing branches of the PV, HA, and bile duct) are located at the six peripheral corners. Blood converges from these triads toward the CV via microvascular channels called sinusoids, while bile flows in the opposite direction.^30^ These sinusoids are specialized; low-pressure capillaries whose microscopic architecture is the smallest repetitive unit of the liver.^31–33^ The liver sinusoidal endothelial cells (LSECs) lining the walls of sinusoids facilitate the transfer of substances between the blood and HCs. In addition, the removal of toxic substances, an essential part of blood purification, is performed by Kupffer cells, a resident type of macrophage found in the sinusoids (Figure 1c). In later sections, we will explore each of these primary cell types. Detoxification, protein synthesis, and metabolic processes in the liver all depend on this particular configuration.^33^

#### 2.1.3 Space of Disse

The Disse space is a narrow area located between the basolateral membrane of HCs and LSECs (Figure 1c). This space plays an important role in the exchange of substances by facilitating the transfer of proteins and other plasma components from the bloodstream to the HCs. This area also contains the microvilli of HCs, which increase the surface area for the exchange.^35^

#### 2.1.4 Liver Zonation

Differences in oxygen, nutrient, and hormone concentrations along the sinusoids lead to the creation of different functional zones (periportal (Zone 1), midzonal (Zone 2), and perivenous (Zone 3) as depicted in Figure 1c. For instance, the partial pressure of oxygen drops from 60–65 mmHg in Zone 1 to 30–35 mmHg in Zone 3.^36^ Different tasks are performed within the liver lobules depending on these concentration gradients. For example, HCs in Zone 1 are involved in gluconeogenesis and urea production, while those in Zone 3 are responsible for glycolysis and drug processing by cytochrome P450 ^37^.

#### 2.1.5 Cell types of the liver

The most common cell types found in the liver are summarized in Table 1 and briefly explained in the next paragraphs.

**Table 1:** Cell Types and Their Key Physiological Features in the Liver

Cell type	In Vivo Abundance (number)	Key Physiological Features	Reference
Hepatocytes (HCs)	60%	Major metabolic unit (CYP450), detoxification, bile synthesis.	^40^
LSECs	∼16–20%	Fenestrated (∼107 nm), porosity ∼6–8% of endothe lial surface. Exchange between blood and cells, filtration.	^21^,^30^,^40^,^41^,^48^
Kupffer cells (KCs)	∼15%	Resident macrophages (immune function); localized mainly in Zone 1.	^40^,^44^
Stellate cells (HSCs)	∼5–8%	Vitamin A storage; ECM synthesis upon activation.	^40^
Cholangiocytes	∼3–5%	Fow and secretion regulation, bile modification, regeneration.	^45^,^49^

##### 2.1.5.1 Hepatocytes (HCs)

HCs are the major parenchymal cells in the liver. They are responsible for the liver’s metabolic activities and protein secretory functions. Compared to nonparenchymal cells (NPCs), HCs are giants, constituting approximately 60% of all liver cells by number yet accounting for about 80% of the human liver’s cellular volume.^38,39^ In terms of isolation yield, healthy human liver tissue yields 20 ± 4 million HCs per gram of tissue.^40^

##### 2.1.5.2 Liver Sinusoidal Endothelial Cells (LSECs)

LSEC constitutes a subgroup of ECs unique to the liver; these cells line the sinusoid walls and are responsible for the efficient bidirectional transport of substances from and to HCs owing to their open pores or fenestrations, which offer a permeable conformation. The detoxification, metabolism, and antimicrobial functions of the liver are facilitated by the unrestricted movement of macromolecules such as plasma proteins via LSEC pores.^32,41^ Additionally, LSECs play a role in immunological defense of the liver by presenting antigens and controlling immune responses.^42^

##### 2.1.5.3 Hepatic Stellate Cells (HSCs)

HSCs are essential for supporting liver tissue regeneration and for regulating connective tissue. Under typical conditions, these cells are located in the Space of Disse wehre they help maintain the extracellular matrix (ECM) stable and store vitamin A. However, HSCs are stimulated in case of liver damage or inflammation, which induces their transformation into myofibroblasts that produce a large amount of ECM components such as collagen. Although this process is essential for liver healing, any overactivation of HSCs can lead to serious liver disorders such as fibrosis and cirrhosis.^40,43^

##### 2.1.5.4 Kupffer Cells (KCs)

KCs play a crucial role in the body’s immune system; they engulf and destroy pathogens, dead cells, and foreign substances detected in blood circulation. Moreover, they modulate inflammatory responses and link the liver with the body’s immune system.^23^ Usually, these immune cells are not arbitrarily spread out; instead, they primarily reside in Zone 1, constituting the first line of defense against possible infections from the intestinal tract.^44^

##### 2.1.5.5 Bile Duct Cells (Cholangiocytes)

Cholangiocytes are epithelial cells that line the bile ducts and are essential for the production and transport of bile, a hepatically synthesized fluid that facilitates fat digestion. Cholangiocytes possess apical primary cilia that act as mechanosensors to detect bile flow and regulate secretion.^45^ Moreover, they modify the bile composition and participate in its reabsorption. Cholangiocytes also facilitate the regeneration of bile ducts during liver damage or inflammation via their fast proliferation.^46,47^

### 2.2 Liver Function

The liver is a multifunctional organ that carries out over 500 essential biochemical processes critical for overall health.^1^ We can categorize the most important functions of the liver under the basic headings of metabolism, storage, secretion, blood purification and xenobiotic biotransformation.^50^ These categories are illustrated in Figure 2, highlighting how the liver engages in various activities that collectively contribute to maintaining body homeostasis.^51^ Modulation of blood glucose levels, bile synthesis, and generation of plasma proteins such as albumin, acute-phase proteins, and coagulation factors are all examples of crucial liver activity.^52^

**Figure 2:**
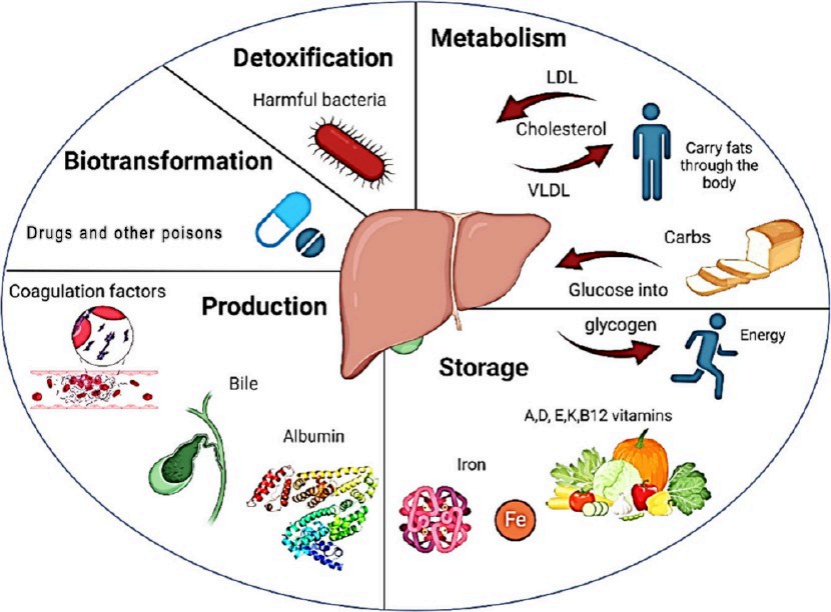
Schematic summarizing the liver functions.

#### 2.2.1 Carbohydrate Metabolism

Controlling the blood concentrations of several metabolites, including glucose and amino acids, is the main metabolic process of the liver; excess glucose is stored as glycogen, which is utilized to perform a glucose-buffering function when blood glucose levels decrease. Furthermore, if needed longer-term, the body uses gluconeogenesis to convert noncarbohydrate molecules such as lactate, glycerol, and amino acids into new glucose.^53^

#### 2.2.2 Protein Metabolism

The liver facilitates amino acid synthesis and conversion of some amino acids into significant chemical substances. It contributes to protein metabolism by forming plasma proteins including albumin, which is crucial for maintaining oncotic pressure and transporting various substances into the blood. The liver also eliminates ammonia from body fluids through urea synthesis.^54^

#### 2.2.3 Lipid Metabolism

In addition to being involved in the metabolism of proteins and carbohydrates, the liver is the site of fatty acid synthesis and oxidation, triglyceride production from fatty acids, and fat synthesis from proteins and carbohydrates. Furthermoe, the liver synthesizes phospholipids, lipoproteins, and cholesterol.^55^

#### 2.2.4 Blood Storage

When blood volume decreases, the liver can supply additional blood, conversely, when blood volume increases, it functions to store excess blood. In fact, the liver, comprising 10% of the body’s blood volume, can contribute an additional 500 to 1,000 mL to the blood reservoir typically present when heart pressure rises.^55^

#### 2.2.5 Preservation of Nutrients

Vitamin A along with vitamins D and B12, are mostly stored in the liver. Other nutrients, including essential minerals, are also preserved in the liver.^56^

#### 2.2.6 Iron Storage

In addition to being a key component of hemoglobin in the blood, iron is also stored in the liver, specifically in hepatic cells, as ferritin.^57^ Ferritin is formed by binding iron to the apoferritin protein, particularly when there is excess iron in bodily fluids. Conversely, ferritin releases iron if the amount of iron in bodily fluids decreases. Thus, this apoferritin-ferritin system functions as an effective iron-storage mechanism, helping to maintain iron homeostasis in the body.^58^

#### 2.2.7 Bile Production

The ability of the liver to secrete bile is one of its vital roles. In the smooth endoplasmic reticulum of HCs, bile is produced as an exocrine secretion in the liver.^59^ Bile acids, bilirubin, cholesterol, phospholipids, and electrolytes are all found in the bile. These materials enter the HCs by absorption from the epithelium, and the HCs then carry them to the bile canaliculi.^60^ Important roles of bile include breaking down and absorbing lipids, as well as removing their breakdown products from the blood.^59^

#### 2.2.8 Blood Purification

Numerous bacteria from the intestinal flora are carried in the blood that emerges from the intestinal capillaries. The hepatic venous sinuses contain Kupffer cells, which filter blood as it passes through and return it clean to the systemic circulation.^39^ These phagocytic macrophages, consume bacteria to digest them effectively.^61^

#### 2.2.9 Drug Metabolic Process

Importantly, the liver is involved in the metabolism of several foreign compounds (xenobiotics), such as drugs and pollutants.^62^ ″Biotransformation″ refers to the chemical conversion of these foreign substances once they enter the body into breakdown products using specific enzymes.^63^ For example, drugs undergo biotransformation, resulting in the production of either active, less effective, or ineffective molecules. Currently, many medications have lipophilic structures that aid absorption. Therefore, biotransformation is necessary for these drugs to acquire hydrophilic properties. Generally, a series of phase I and phase II reactions are involved in xenobiotic metabolism: Phase I activation events include oxidation–reduction and hydrolysis, mediated by monooxygenases dependent on cytochrome P450 (CYP450) enzymes.^64^ As a result, free radicals and hazardous compounds are generated.^64,65^ On the other hand, phase II controls conjugation with sulfate, glycine, glucuronic acid, glutathione, and acetate to induce detoxification processes. Glucuronidation is the primary reaction in this phase which makes the medications or their metabolites water-soluble for excretion through urine or bile.^66^ When the equilibrium between the activation and detoxification processes is disturbed, hepatotoxicity can occur.

### 2.3 Liver Diseases

Liver diseases cause two million deaths each year, accounting for 4% of total deaths (1 in every 25 deaths worldwide). Among these, liver cancer alone is responsible for 600,000 to 900,000 deaths; making it the 11th leading cause of death,^67^ though liver cancer-related deaths could potentially be underreported.^68^ To understand their extent, this section will explore major liver diseases and highlight the importance of modeling them.

#### 2.3.1 Alcoholic and Nonalcoholic Fatty Liver Diseases (ALD and NAFLD)

Ethanol, the leading cause of cirrhosis globally,^67^ is oxidized in the liver by alcohol dehydrogenase (ADH) enzymes to acetaldehyde, a recognized carcinogen and a frequent cause of ALD, particularly after excessive consumption of alcohol. This process can lead also to the formation of adducts, which are chemical alterations that disrupt normal biological functions.^69^

On the other hand, NAFLD, also called MAFLD or MASLD, is driven by an unhealthy diet and low level of activity, affecting 30% of the general adult population, making it one of the most prevalent chronic liver disease globally.^67,70,71^ This illness progresses in two stages: first, fat accumulates in hepatocytes as the form of triglycerides. This buildup induces hepatocyte injury and inflammation, known as nonalcoholic steatohepatitis (NASH), whose mechanism is not fully understood but likely involves oxidative stress and activation of proinflammatory cytokines, ultimately resulting in fibrosis, which is the excessive accumulation of scar tissue.^72,73^ Unfortunately, NAFLD presents heterogeneously among patients,^74^ underscoring the importance of developing personalized models and effective targeted therapeutics.

#### 2.3.2 Hepatitis B Virus (HBV) and Hepatitis C Virus (HCV)

HBV and HCV are chronic infections transmitted through the blood, often due to needle sharing or infected objects.^75,76^ HBV is a compact DNA virus that shares some characteristics with retroviruses, and affects approximately 300 million people worldwide.^77^ Little is known about nuclear proteins encoding its cccDNA, which must be silenced or eliminated to cure HBV.^78^ Both HBV and HCV can lead to insulin resistance and worsen steatosis by increasing oxidative stress, which is also observed in NAFLD and ALD. However, the specific processes involved are not completely understood. Currently, universal antiviral medications and direct-acting antivirals (DAAs) that affect the replication mechanism are the standard in the medical treatment of HCV, but can lead to potential risks for hepatocellular carcinoma (HCC).^69^ On the other hand, there is no cure for hepatitis B,^79^ highlighting the crucial role of modeling in understanding and treating these diseases. Exploring the genes responsible for altering liver function through relevant models, which enables the measurement of protein expression and transcriptomic changes in transgenic cells compared to healthy cells, is critically important for this field of research.^80^

#### 2.3.3 Cirrhosis

All of the previously explained diseases can lead to cirrhosis, with ALD being the most common.^81^ Cirrhosis is a late-stage condition characterized by regenerative nodules, fibrous bands, and vascular distortion due to chronic injury. The severity of liver cirrhosis worsens as more scar tissue builds up, causing direct disruption to liver function and eventually leading to gradual liver failure and potential death.^82^

#### 2.3.4 Hepatocellular Carcinoma (HCC)

HCC, which can develop from cirrhosis, is the third-leading cause of cancer death worldwide.^83^ Improper management of ALD, NAFLD, HBV and HCV may also progress to HCC.^80^ The molecular pathways involved in these transitions are not yet fully understood.^84^ During the initial stage, potential cures, such as surgery, liver transplantation, and local ablation, can enhance the patient’s chance of survival. However, this illness is usually detected at a late stage. Additionally, certain currently available treatments are limited to palliative care and localized treatment. Early detection of HCC together with appropriate treatment are essential for boosting survival rates and enhancing the quality of life of patients. Therefore, researchers are investigating novel biomarkers with high sensitivity and reliability,^85^ where relevant preclinical models are greatly needed.^86^ Relevant microphysiological systems (MPS) that accurately replicate HCC pathophysiology can also be very useful in studying tumor heterogeneity and resistance mechanisms,^87^ as well as in developing patient-derived therapies.^88^

#### 2.3.5 Liver Drug-Induced Injury (DILI)

another serious disease characterized by liver impairment triggered by certain drugs and their metabolites.^89^ DILI is one of the main causes of acute liver failure (ALF) and liver transplantation worldwide.^90^ It was also the leading cause of postmarketing withdrawals of 462 medications between 1953 and 2013.^91^ The low accuracy of traditional methods, such as 2D in vitro and animal models, is mainly responsible for the low predictability of DILI during the preclinical phase.^92,93^ Several hazardous drugs and their metabolites can damage the mitochondria, induce oxidative stress, and affect liver cells through various molecular pathways, such as direct hepatotoxicity and immunological reactions. Nevertheless, the pathophysiology of DILI is very complicated and still poorly understood.^89^ Furthermore, a recent type of DILI has been identified and needs to be treated using nontraditional methods, with the number of cases anticipated to increase.^90^ For the aforementioned reasons, the development and implementation of highly predictive models are critical to both exploring the mechanisms and the complex interactions implied in the genesis of DILI, as well as to enhance and expedite the development of novel therapies.

### 2.4 Importance of Liver-on-a-Chip (LoC) in Liver Modeling

The pharmaceuticals development process is labor-intensive and costly. It takes approximately 12–15 years from target identification to marketing approval.^94^ According to an economic evaluation study, the estimated median R&D cost was $1.1 billion per drug for those approved by FDA between 2009 −2018.^95^ Prior to their introduction into human clinical tests, lead candidate drugs usually pass through an evaluation of their adsorption, distribution, metabolism, excretion, and toxicity abbreviated as ADMET, and tested in traditional *in vitro* and *in vivo* (animal) models.^2^ However, more than 90% of drug candidates fail in clinical trials,^96^ with hepatotoxic effects undetected in preclinical testing frequently contributing to these failures.^97^ The research of NAFLD disease and treatments is also still based on rodent models, despite several notable differences between animals and humans, including the mechanisms of adipose accumulation, the severity of liver fibrosis, and other factors.^98^ Consequently, several human-relevant in vitro liver models have been developed. In this context, OoC technology presents the hope of replacing, reducing, and refining (the “3Rs”) the use of animal models, which is especially essential for LoC because most studies and tests related to the liver still rely on animal models.^15^ This shift is necessary not only for improving human relevance, but also for addressing ethical issues related to animal testing, as already clarified in the introduction. LoC could furthermore lower the R&D time and cost. For example, Atkins et al. were able to screen 35 novel lipid nanoparticles (LNPs) using a commercial LoC model during a course of experiments that took 18 months at a total cost of $325,000—4 times faster and at 6.5% of the cost of using animal models.^99^

#### 2.4.1 But What Is Specific about LoC Compared to Other In Vitro Models?

The traditional in vitro model is generally a 2D static culture of primary human hepatocytes (PHHs) or human hepatoma cell lines, largely used since the 1900s because of its simplicity, low-cost maintenance, and ease of operation.^100^ However, it only permits cells to grow in a flat monolayer, gradually become depleted of nutrients, and morphologically altered, thus having much lower physiological relevance. On the other hand, 3D static culture (on transwell inserts, scaffolds, or spheroid culture plates) produces more physiologically relevant models as they ensure cell–cell interactions and extracellular matrix (ECM) communication, but they also suffer from poor nutrient transport due to the lack of fluid flow.^15,101^

To overcome these shortcomings, multidisciplinary researchers have innovated an advanced MPS termed LoC with engineered microchannels that enable blood-simulating cell culture medium (CCM) to precisely perfuse cells with a physiologically relevant flow rate. Microfluidic technology, which plays a pivotal role in various biomedical applications,^102–106^ is also a key aspect in these organ-chip systems; it allows for the precise manipulation of fluids in channels at the microscale to replicate complex biological processes. Unlike conventional 2D and 3D static culture, the provided dynamic flow enables a continuous delivery of nutrients, chemicals (e.g., drugs), and signaling molecules which are crucial elements of the hepatic microenvironment, enabling accurate simulation of the liver.^107,108^ Further than ensuring the supply of growth factors and oxygen along with the removal of waste, these microfluidic systems provide cultured cells with shear force, which helps maintain their phenotypes for extended periods and enables functions that elicit responses more closely aligned with in vivo settings.^109–112^ Moreover, LoC technology offers great flexibility, allowing the construction of different models with various designs and cell configurations. In addition to the mechanical microenvironment, advanced models can provide multiple cell–cell interactions and spatiotemporal constructs to further enhance the mimicry of liver microarchitecture and physiology. In the following sections, we elucidate recent LoC models developed by scientists as well as by industry experts.

## 3 Case Studies of Academic LOC Models

### 3.1 Monocellular Models

In practice, the simplest in vitro LoC models employ monocellular systems based on traditional cell-culture methodologies. The designs of these models can be very primitive, consisting of only one channel, such as the chip of Li et al.^113^ used to culture PHH. Despite its simplicity, this chip could—compared with standard well plates— provide the key physiological responses in the hepatotoxicity of SPION nanoparticles due to the flow provided.

Other models were relatively more complex but were designed to focus on replicating a specific phenomenon or component of the liver, such as acinus zonation and the architecture of sinusoids or lobules.

Ghafoory et al.^114^ addressed the O_2_ zonation concept by serially connecting four microfluidic chips to seed and cultivate HepG2 cells, where their oxygen consumption created a hypoxia gradient. More recently, Mahdavi et al., proposed a more compact design comprising a HepG2 chamber with oxygen diffusing from two nonintersecting gas channels (having high and null O_2_ levels) engineered to generate three distinct oxygen zones (Figure 3a). Kang et al.^116^ further modeled metabolic zonation within an LoC device, which consists of a primary hepatocyte chamber and a microfluidic gradient generator with two orthogonal axes for patterning flows (Figure 3b). Although this model appears complex, it permits the generation of stable gradients for different metabolic modulators.

**Figure 3:**
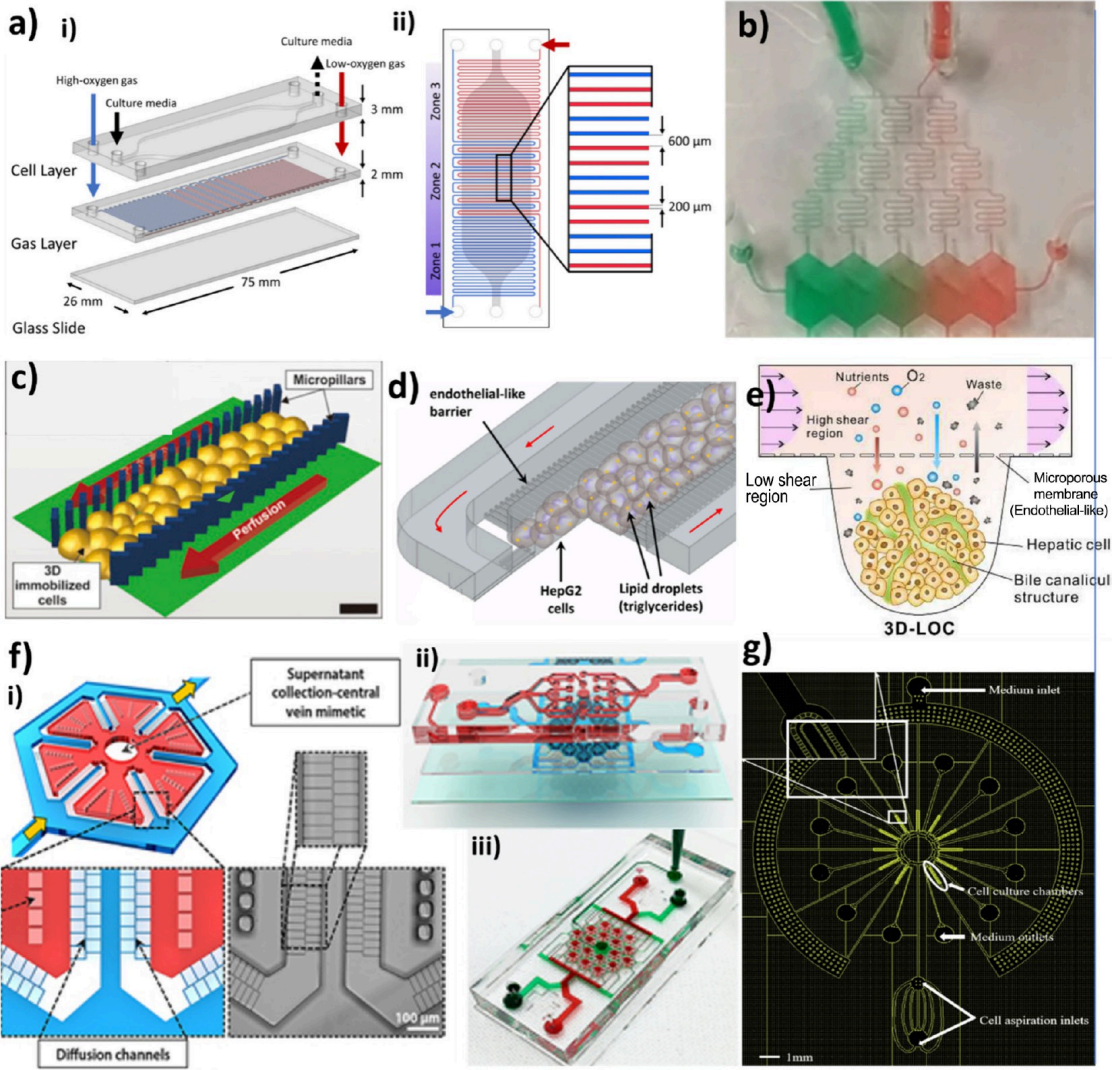
Examples of academic monocellular LoC models a) Design of an O2-zonation-based model: (i) split view and (ii) top view, reprinted from ref 115 Copyright 2025 Springer Nature, under the open access license Creative Commons Attribution 4.0 International (CC BY 4.0) license. b) Gradient of food dye illustrating the design of a model focused on metabolic zonation, reprinted from ref 116 and licensed under CC BY 4.0, Copyright 2018 Springer Nature. c), d) and e) Sinusoid-inspired models c) used micropillars to create a perforated barrier, reprinted from ref 117. Copyright 2017 Wiley d) used micro cuboid-shaped barriers, reprinted from ref 118 and licensed under CC BY 4.0. Copyright 2016 PLOS. e) Schematic of an LoC model to culture spheroids on V shaped wells protected by a microporous membrane that mimics the fenestrated endothelial barrier, reproduced from ref 34 with permission from the Royal Society of Chemistry. f) VLSLL-on-a-chip: Schematics of (i) lobule-like chamber exhibiting PDMS pillars, (ii) top seed-feed network aligned on the bottom layer incorporating 18 single-lobule mimetic chambers, (iii) physical image of the assembled chip, reprinted from ref 119. Copyright 2017 IOP. g) Design of another lobule-inspired model, reprinted from ref 120. Copyright 2024 Wiley.

Other researchers focused in developing sinusoid-inspired LoC monocellular models, where the endothelial barrier with its LSECs’ fenestrations was mimicked by a physical barrier perforated by parallel microchannels,^117,118^ ensuring diffusion-dominated nutrient transport from perfusion channels to the central cell culture compartment without a direct flow to the HCs, thereby protecting them from high shear forces. Models based on this perforated barrier principle were employed to differentiate 3D HepaRG spheroids^117^ (Figure 3c) as well as to culture HepG2 cells under free fatty acid supplementation to model NAFLD^118^ (Figure 3d), where both showed significant efficiency and reliability compared to their control 2D cultures. Similar to the perforated barrier, Ma et al.^34^ inserted a PET porous membrane to separate the lower cell culture channel from the upper flow channel to avoid the negative effects of high shear stress (Figure 3e). Using micromilling technology, an array of V-shaped concave microwells at the bottom of a long culture channel was fabricated and used to seed HepG2/C3A cells and help them aggregate into 3D spheroids over time. This model significantly improved the polarization, liver-specific functions, and metabolic activity of spheroids, demonstrating better long-term maintenance compared to conventional methods.

However, more models have been built based on the perforated barrier concept to mimic the entire lobule, instead of a single sinusoid. Banaeiyan et al.^119^ fabricated a large-scale liver-lobule (VLSLL)-on-a-chip device consisting of hexagonal culture chambers separated by perforated walls from a seed-feed network (Figure 3f). Each perfusion network had a central outlet that mimicked the central vein. In addition, polydimethylsiloxane (PDMS) pillars have been engineered to support large tissue culture chambers, facilitating the attachment and radial alignment of newly seeded cells. This device was tested with HepG2 cells and hiPSC-derived HCs. Despite being complex, it demonstrated high physiological relevance, stable albumin secretion, urea synthesis, and bile canaliculi formation. Furthermore, the cells maintained excellent functionality and morphology during long-term culture. Recently, Rajendran et al.^120^ designed a similar but much simpler system to mimic 3D hepatic cords organized radially, similar to lobules (Figure 3g). HepaRG - hepatoblasts (HepaRG-HB) were able to proliferate, self-organize, and spontaneously differentiate in the device culture chambers, forming long, directional bile canaliculi.

Although monocellular models are relatively simple and have been applied in many aspects, their lack of physiological mimicry leads them to fail to capture the critical cell-to-cell interactions and complex microenvironments that are essential for accurate tissue function and reliable responses of the liver in vivo. Therefore, several LoC multicellular models have been developed and are reviewed in the next section.

### 3.2 Multicellular Models

#### 3.2.1 Importance of Cell–Cell Interactions

Cells interact with their niche environment as well as with many temporary and permanent cells within it. These interactions are vital for cells to fulfill their various functions. In the liver, interactions between parenchymal and nonparenchymal cells (NPCs) have been reported to trigger cell migration, differentiation, proliferation, and apoptosis. The formation of endodermal foregut and mesenchymal vascular structures in the liver is believed to be also mediated by these heterotypic cell–cell interactions.^121–123^

Different types of liver cells communicate among themselves, with each other and with their environment through various signaling pathways. These signals involve paracrine and autogenic interactions between various mediators including prostanoids, nitric oxide, TNF-α, interleukins, chemokines, growth factors, and reactive oxygen species. For instance, liver stellate cells regulate sinusoidal blood flow and various growth factor levels, while Kupffer and stellate cells modulate the cytokines which influence the secretion of IGF-1, a growth factor primarily produced by the liver.^124^ This intricate signaling network also affects metabolic processes. For instance, inflammatory mediators such as prostaglandins affect glucose metabolism by modulating glycogenolysis in HCs, while leukotrienes, also synthesized by Kupffer cells, further influence liver function.^125,126^ Under certain pathological conditions, this complex crosstalk may induce some diseases such as endotoxemia and liver fibrosis. Membrane structure changes in both healthy and pathological liver cells represent one these conditions, as various studies emphasized its role in fibrosis.^127^

Slevin et al.^128^ further explored how cellular interactions shape disease progression by investigating the effects of prolonged alcohol consumption on ALD. Their research findings revealed that multiple cell types contribute to the progression of ALD, with liver macrophages, particularly KCs, playing a predominant role. KCs were found to contribute to the development of ALD by producing various chemokines and cytokines that promote cell–cell contact. Therefore, understanding liver cell cooperation is vital for understanding both normal physiological and pathological processes.^129–131^

The invention of LoC technology has received significant attention in literature, highlighting the significance of cell–cell interactions in research. Although the exact mechanisms by which NPCs alter hepatocyte phenotypes are yet unknown, understanding the importance of cell signaling is essential for studying diseased livers in vitro. Current developments in cell culture and microfluidics not only facilitate the creation of functional tissue structures for medical applications, but also offer opportunities to gain deeper insight into how cell communication functions in physiological and pathological processes.^123^ In this context, advanced LoC models are essential tools for the study of cell–cell communication, not only because they successfully replicate the microarchitecture of the liver and enable the culture of HCs, but also because they allow these cells to be cultured alongside NPCs that e likewise constitute the liver.^110–112^

For instance, Du et al.^109^ simulated lipopolysaccharide (LPS) infection using a two-channel LoC model that integrated four types of primary liver cells. Their findings revealed that LPS triggers the adherence of neutrophils (a type of white blood cell) to liver sinusoidal endothelial cells (LSECs) during cell recruitment. The researchers reported a 63% increase in neutrophil accumulation, demonstrating that coculture LoC platforms are highly suitable for investigating cell–cell interactions.^109,132^

Another study explored the interactions between HSCs and HCs in 3D culture, but without direct cell–cell contact, using an LoC platform with concave microwells. According to Lee et al., spheroids are more likely to form when continuously exposed to flow. They further demonstrated that HSCs help form and maintain hepatocyte spheroids, where interactions between cells enhance the characteristics of these spheroids, including their size and physiological responses. Additionally, compared with conventional static culture systems, the amounts of albumin and urea produced in this 3D culture within the LoC were remarkably increased.^133^

Details on various academic LoC models that benefit from these cell–cell interactions are provided in the following sections, exploring different designs and configurations.

#### 3.2.2 Models Mimicking Sinusoids

As explained in Section 2.1, the liver sinusoid, constituting the liver’s basic repeating microscopic unit, contains various types of hepatic cells organized in a controlled environment to maintain hepatic functions. By incorporating microengineering techniques, up to four primary hepatic cell types can be combined, for instance, in a two-layer system with shear flow, mimicking blood flow and interstitial flow in the sinusoid.^7,109,134^ Such models enhance liver-specific functions and replicate immune responses such as neutrophil recruitment under stimulation.^135–137^ These liver sinusoid-chip systems serve as *in vitro* models to also enable studying short-term cellular interactions within a miniaturized physiological liver environment.

##### 3.2.2.1 Mechanical Microenvironment of Hepatic Sinusoids

The microenvironment in the liver sinusoids is highly complex, which obstructs the development of in vitro models intended to replicate them. Their unique vascular structure enables slow blood flow, estimated to range at 407–451 μm/s, and regulated through the LSECs fenestrations measuring interstitial flow presents considerable challenges.^138^ This dynamic flow affects hepatic metabolic processes due to the induced shear stress. On the other hand, ECM plays a crucial role in various processes, including cellular mobility. Another important feature is the liver stiffness, which typically ranges between 400 and 600 Pa under normal conditions, while it rises up to 1.2 to 1.6 kPa in fibrotic liver.^138–140^ Notably, mechanical heterogeneity has been observed between regions such as the periportal area and central liver. Thus, hepatic cells able to perceive and respond to their microenvironment signals, thereby activating intracellular signaling pathways.^138^

This intricate interplay of blood flow, ECM structure, and mechanical heterogeneity underscores the challenge of accurately mimicking the liver sinusoids in vitro. LoC systems address this more effectively than other MPSs by providing the necessary fluid flow, stiffness, and shear stress conditions together with multicellular culture, thereby offering a more precise replication of the sinusoidal microenvironment, which also enable systematic investigation of LSEC mechanobiology.

Although LSEC behavior is known to be regulated by shear stress and mechanical strain from microenvironment stiffness, the comprehensive effects of mechanobiology on these cells remain underexplored.^141,142^ To fill this gap, LoC sinusoid models have been employed: one study revealed that increased shear stress LSEC boosted nitric oxide production. Another recent study highlighted the significance of mechanosensitive pathways in LSECs by studying the impact of pathological pressure on the their phenotype, revealing the role of the LoC model used in identifying pressure-related genes as genomic biomarkers of portal hypertension.^143^ The team developed a fluidic device called Exoliver that provides fluidic stimulation to the top layer where LSECs were cultured on a hydrophilic polytetrafluoroethylene (PTFE) microporous membrane under continuous shear stress, while HCs were plated in the lower poly­(methyl methacrylate) (PMMA) compartment. The dynamic configurations of Exoliver began with a shear stress stimulus of 0.1 dyn/cm^2^, gradually increasing to 1.15 dyn/cm^2^ (1.5 mL/min) over the first 2 h (Figure 4a). The Exoliver, along with the reservoir, filters, and most of the tubing, was placed inside an incubator to maintain physiological conditions (37 °C, 5% CO2).^144^

**Figure 4:**
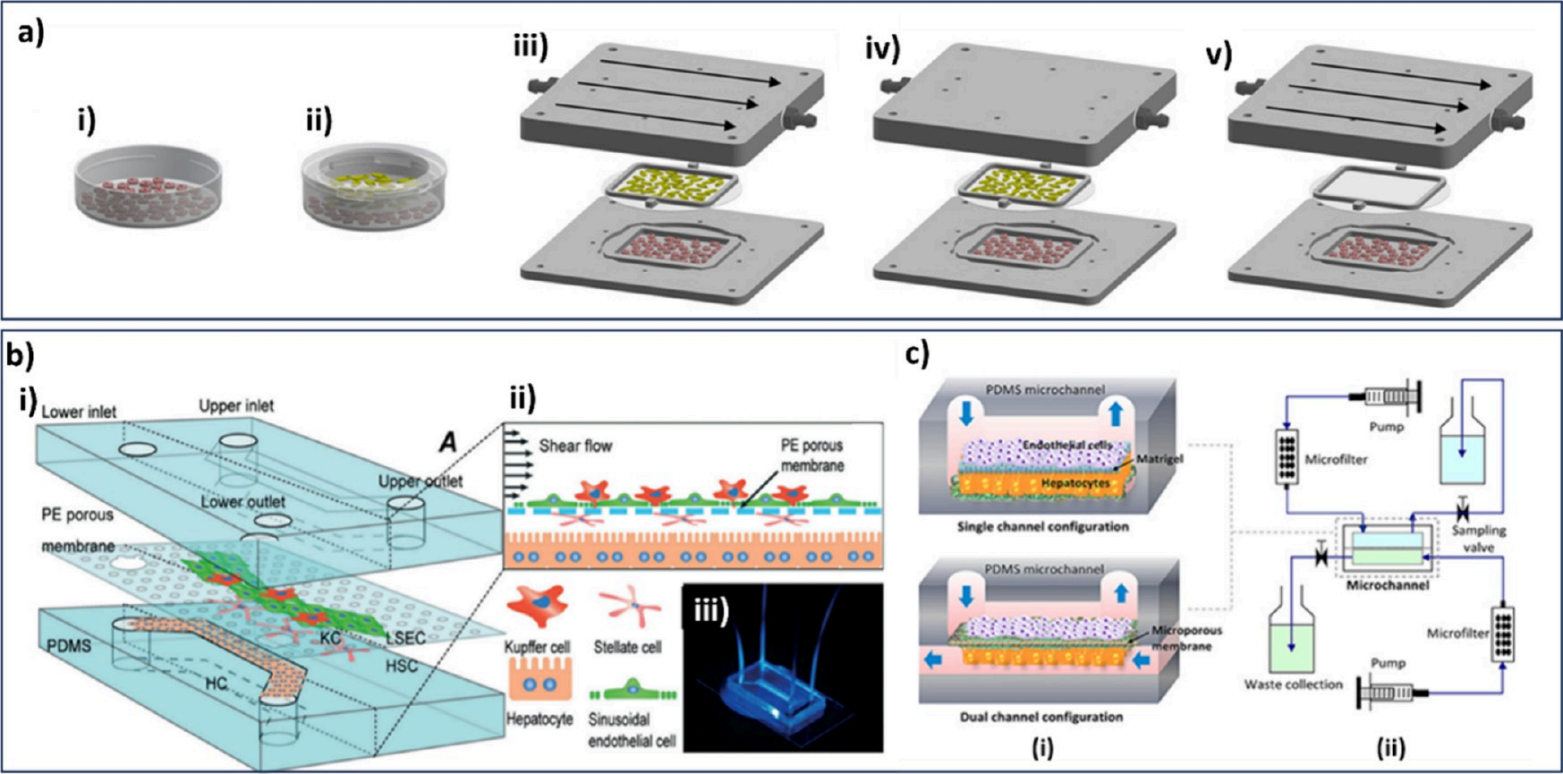
a) Comparison between in vitro conventional culture methods and those with Exoliver design: (i) HCs monoculture in Petri dish. (ii) Coculture (HCs and LSECs) in transwell. (iii) Dynamic coculture system within Exoliver, stimulated with continuous and homogeneous shear stress (optimal condition). (iv) Static coculture within Exoliver without shear stress, leading to LSEC dysfunction and (v) Dynamic monoculture system, reprinted from ref 144 and licensed under CC BY Copyright 2018 Wiley. b) Diagram of a 3D sinusoidal LoC model. (i) microfluidic PDMS chip PDMS with collagen-I coated polyester membrane. (ii) 3D assembly of four types of cells: LSECs, KCs, HSCs, and HCs. (iii) Photographic image of the chip, reprinted from ref 109 with permission from the Royal Society of Chemistry. c) Other sinusoid-like LoC platforms comprising (i) single-channel and dual-channel microfluidic configurations; and (ii) a bioreactor circuit for continuous media perfusion and waste collection, reprinted from ref 145. Copyright 2015 Wiley.

The results of these studies also indicated that primary human and rat HCs cocultured with shear stress-stimulated LSECs within Exoliver maintained their morphology and exhibited high albumin and urea production. These HCs further demonstrated enhanced CYP3A4 activity and sustained expression of HNF4α and its transporters, indicating a delayed dedifferentiation. Besides, HCs cultured in this liver-mimicking device showed significantly different responses to acute treatment with known hepatotoxic drugs than those cultured on conventional platforms.^144^

While physiological shear stress in LoC systems preserves LSEC phenotype, fibrotic livers impose pathologic tissue hardening that reverses these beneficial mechanosensitive responses; increased ECM deposition and stiffness lead to higher vascular resistance and activation of hepatic stellate cells (HSCs), while simultaneously causing podosome formation and dedifferentiation in LSECs that reduce their ability to produce vasoactive mediators, resulting in capillarization. Recent studies have suggested that restoring LSECs to a nonstiff environment can improve their phenotype, offering potential for new therapeutic approaches.^139,141^

##### 3.2.2.2 Liver-on-a-Chip Sinusoidal Constructs

These LoC models involve coculturing different cell types with HCs, such as LSECs, and macrophages, as well as incorporating shear flow to enhance liver functions, such as urea secretion and protein synthesis.^109,145,146^ The inclusion of these NPCs is essential for studying the overall hepatic responses and drug toxicity. Developing better liver sinusoid chips requires isolating and incorporating purified NPCs into these models.^7^

Generally, these models use microfluidic channels lined mainly by LSECs, together with other NPCs, to replicate the liver’s sinusoidal microvascular structure.^142,143,145,147,148^ Many liver diseases are known to be associated with disruption of the functional structure of the liver sinusoids. However, the pathophysiology remains incompletely understood because of their complex structure. Innovative LoC models that resemble sinusoids are being developed, providing opportunities to understand sinusoidal biology, disease development and progression, and the processes involved in drug responses.^141,143,148^

Du et al.^109,138^ developed one such model using a 3D microfluidic configuration (Figure 4b). This chip integrated four types of primary hepatic cells into two fluid channels separated by a permeable membrane. Computational fluid dynamics simulations were used to investigate the flow field by analyzing the velocity profiles and liver-specific functions. Particle tracking visualization tests were conducted to estimate the particle velocity and wall shear stress. The presence of the LSECs was found to influence the flow velocity within the chip. The production levels of albumin (ALB), urea, hepatocyte growth factor (HGF), and vascular endothelial growth factor (VEGF) were measured and the activities of cytochrome P450 (CYP) enzymes were also evaluated. The results reveal that shear flow and coculture with NPCs influenced the secretion of ALB, the production of HGF and the activities of CYP enzymes. Overall, this liver-chip successfully replicated the physiological features of a liver sinusoid and proved the synergistic effect of cell coculture and fluid flow on liver functions.

A similar study introduced a microfluidic platform for coculturing primary rat HCs and LSECs, replicating the liver sinusoid structure using single- and dual-channel systems with a porous membrane (Figure 4 c). The dual-channel platform with continuous perfusion successfully maintained long-term coculture for over 30 days, preserving hepatocyte functions, such as urea production.^145^ From our perspective, the two-channel configuration is more effective because it better represents the space of Disse’s structure, —thus increasing the physiological fidelity compared to the single-fluid arrangement.

Another team developed another LoC model featuring a single microchannel with micro fences. HCs, HSCs, and LSECs were sequentially layered within the central microchannel, creating a sinusoid-like structure regulated by laminar flow. KCs were further positioned on the surface of the LSEC layer. Adjacent to the central microchannel are additional microchannels designed for the perfusion of artificial blood and bile flowing oppositely. This model demonstrates superior hepatic activity (cell viability, albumin synthesis, urea secretion, and cytochrome P450 enzyme activities) compared with traditional planar cell culture models.^149^ This study underscores the significance of investigating the interactions between LSECs and vascular targeting in hepatocellular carcinoma (HCC).

Another work introduced a coculture model (SK-HEP-1 and HepG2/C3A spheroids) within an integrated insert on a dynamic microfluidic platform (IIDMP) illustrated in Figure 5 to mimic the space of Disse. This model used a hyaluronic acid hydrogel scaffold to hold the spheroids. The performance of this model was assessed by measuring PECAM-1 and stabilin-2 expression levels and evaluating barrier permeability and transport properties. The study findings indicate that the SK-HEP-1 cell layer, a surrogate for the primary LSEC barrier, formed much denser tissue under dynamic coculture conditions, while maintain the HCs morphology and functionality for long-term.^147^ Importantly, although the system used in this study does not rely on microfluidic channels, it illustrates how simpler adaptations—such as plate wells with permeable inserts under dynamic setups—can help mimic the sinusoidal functions, elucidate endothelial-hepatocyte crosstalk and investigate drug metabolism.

**Figure 5:**
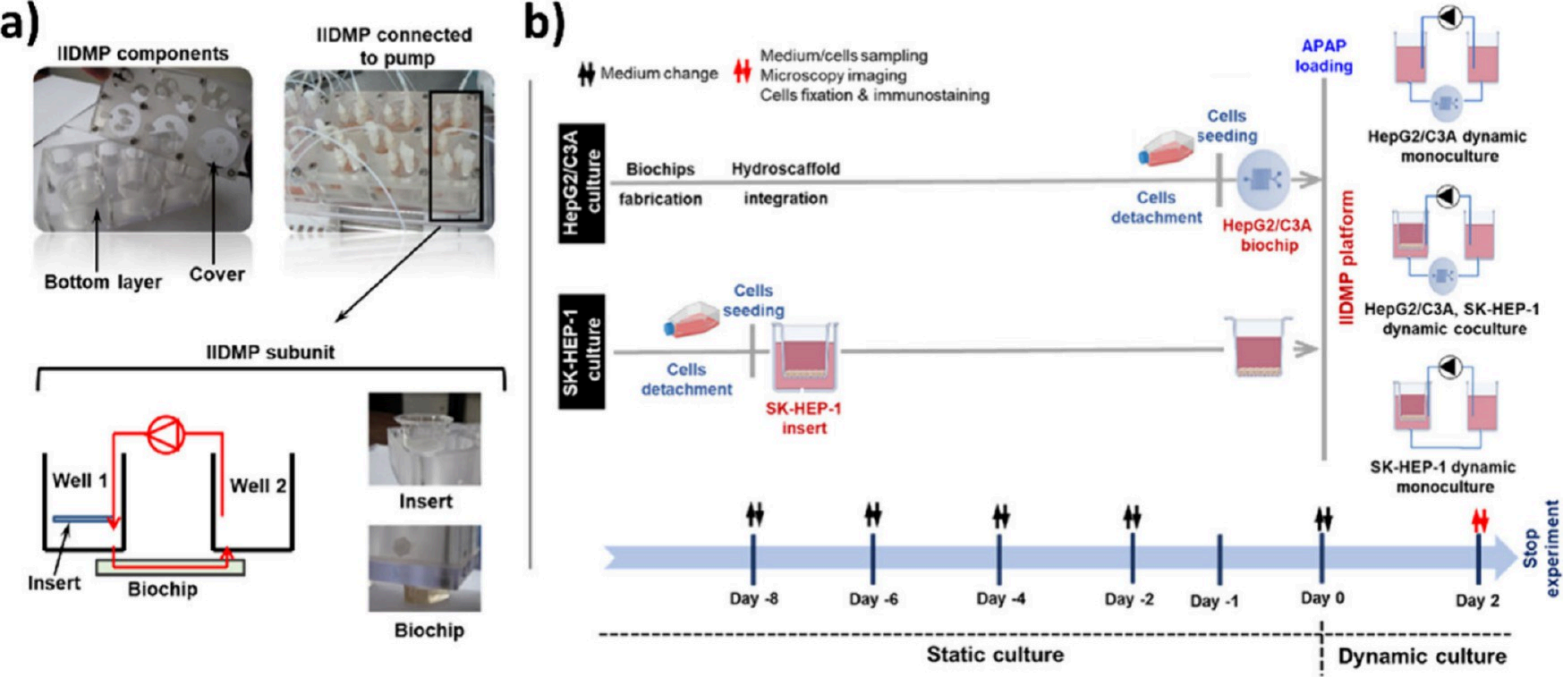
(a) Images and diagrams show the IIDMP coculture platform design, which features a polycarbonate structure with three compartments facilitating culture medium exchange between the LSEC barrier and hepatocyte biochip. (b) Details of the experimental procedures for SK-HEP-1 and HepG2/C3A in both the monoculture and coculture setups. Each dynamic experiment lasted 2 days, with SK-HEP-1 inserts cultured for 8 days to form a confluent barrier before starting the experiments, reprinted from ref 147. Copyright 2024 Elsevier.

A recent study by Li et al. reported the design and operation of a hepatic acinus chip (mHAC) (Figure 6). This study focused on large-scale manufacturing of liver-chips using microneedle arrays to fabricate liver-acinus-chip microsystem with a dual blood supply. Microneedles with different radii, designed using SolidWorks and fabricated using a 3D printer, were employed to create primary sinusoids to enhance the metabolic function of HCs. Differentiated HepaRG cells and Human LSECs were mixed with GelMA, which was used as the ECM. The porosity value was calculated to match that of human liver tissue. The results revealed that concentrations of biomarkers and activities of enzymes were significantly higher in the microneedle-assisted groups. The study showed that chips with induced sinusoids promotes much higher cell viability compared to uninduced groups, specifically after optimizing the needle radius. Interestingly, secondary sinusoid formation was observed, which helps deliver nutrients to cells located farther away from the primary sinusoids.^150^ Therefore, this model provides a highly advanced platform due its scalable replication of acinar vascular architecture with multiple primary and secondary sinusoids.

**Figure 6:**
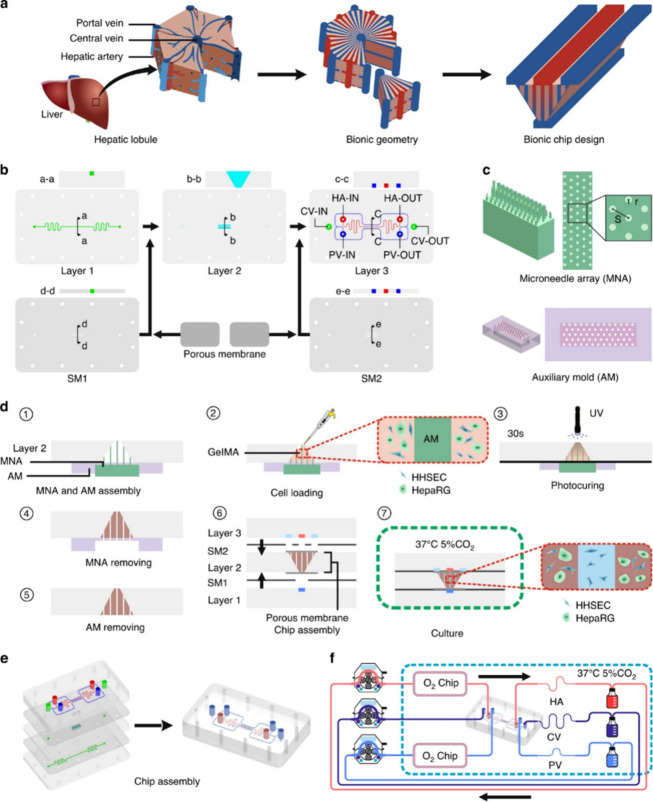
Design of the mHAC: Diagrams of a) hepatic acinus showcasing the trivascular system b) components of the mHAC. c) Microneedle array and its associated mold for demolding. d) Assembly process of the mHAC. e) 3D representation of the chip. f) Experimental setup for the mHAC, with the wavy line representing the polytetrafluoroethylene (PTFE) tube and the straight line representing the silicon tube reprinted from ref 150 and licensed under CC BY. Copyright 2023 Nature.

#### 3.2.3 Models Mimicking Lobules

The lobule has a distinctive hexagonal architecture and constitutes a repetitive anatomical unit of the liver (See Section 2.1). Several LoC models have been established with the aim of replicating the structure, composition, and function of hepatic lobules, referred to as liver lobule chips (LLCs). Typical techniques used for the construction of these LLCs include the use of traps and micropatterning approaches to capture and arrange cells.

For instance, Ma et al.^151^ constructed a 3D lobule-like liver microfluidic chamber in which HepG2 cells and immortal human aortic endothelial cells were loaded into the desired positions using an integrated pneumatic microvalve system, then immobilized, arranging them in a radial pattern of multiple pillar arrays. This LLC replicated the radial arrangement of the hepatic cords and the intrinsic network of hepatic sinusoids (Figure 7a). The microtissue produced showed elevated levels of baseline CYP-1A1/2 and UGT activity, dynamic responsiveness to enzyme induction/inhibition, and a significant capacity for drug metabolism in the liver. This study effectively analyzed the potential adverse medication responses causing liver damage, revealing that preadministered medications that stimulated CYP-1A1/2 and/or UGT activity affected the toxic response. From our analysis, the effectiveness of this model in toxicological investigations was confirmed. Furthermore, its bioengineering approach which combines hydrodynamic and microstructure-guided patterning offers advantages for liver tissue engineering, liver physiology and pathology studies, and drug-induced hepatotoxicity evaluation.

**Figure 7:**
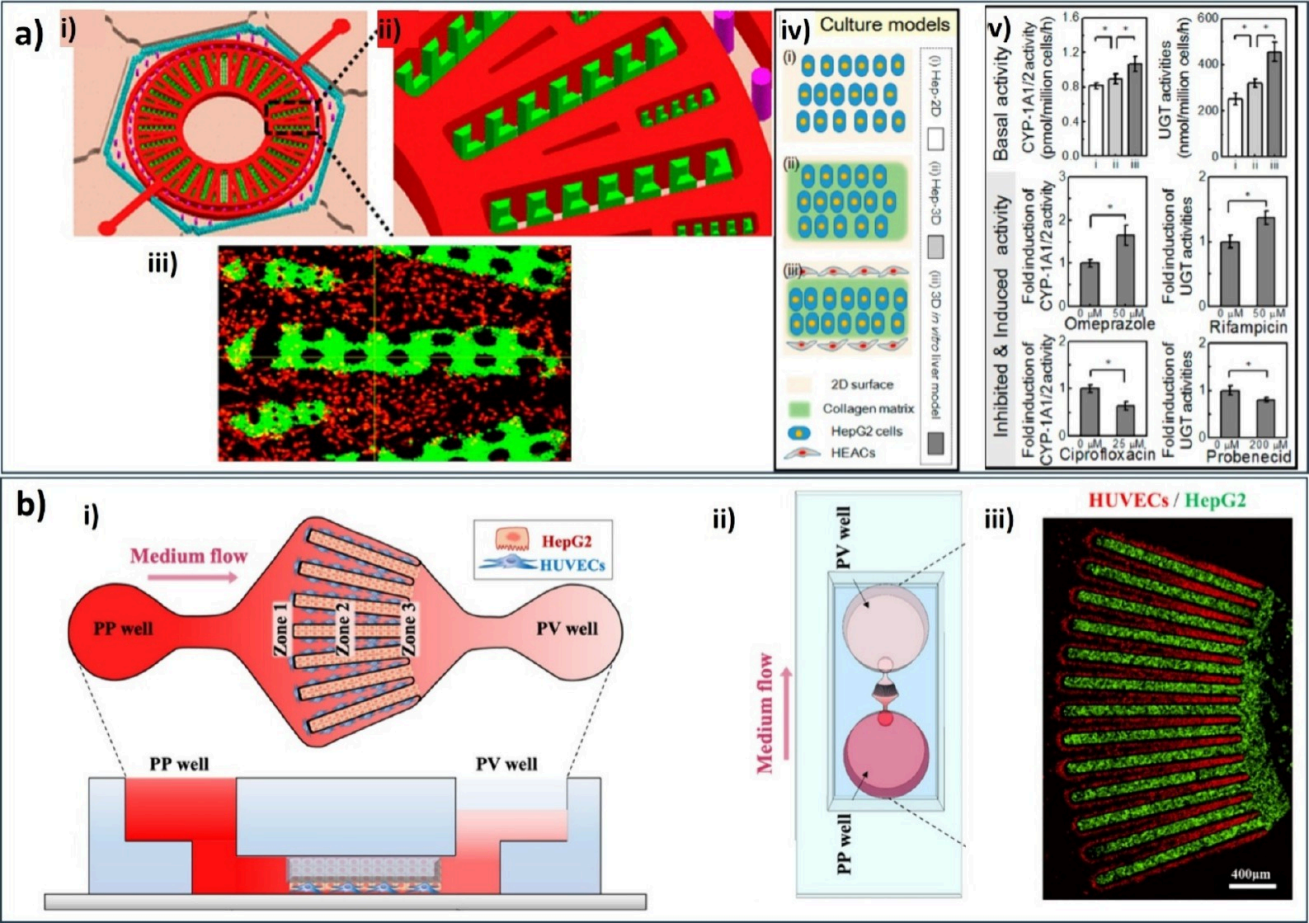
a) LLC schematic. (i) and (ii) 3D configuration showcasing a hexagonal cell culture chamber with radially micropatterned pillar arrays and a pneumatic system. (iv) Development of three distinct culture models in the LLC and (v) Assessment of hepatic enzyme activity, adapted with permission from ref 151. Copyright 2016 American Chemical Society. b) Schematic of LADY model. (i) Details coculturing: HUVECs were injected into the PP well while HepG2 cells applied into the PV well, trapped by a weir structure and arranged in a partial radial pattern. (ii) Top view of the chip. (iii) Cocultured RFP-expressing HUVECs and GFP-expressing HepG2 cells for the morphological demonstration of LADY operated for 12 days, adapted from ref 152 and licensed under CC BY 4.0. Copyright 2022 MDPI.

Kwon et al. focused on developing a chip model of the liver acinus, a repetitive component of the liver lobule. This device facilitated hepatic zonation through the establishment of a gradual flow and enabled 3D coculture of HepG2 with HUVECs (Figure 7b), resulting in a gradient of drug-induced hepatotoxicity across the culture chamber.^152^ In the reviewers’ perspective, this liver acinus dynamic (LADY) chip is one of the few models that recapitulate liver functional zonation enabling the precise assessment of zonal hepatotoxicity. Moreover, its unique weir structure allows for effective coculture of HCs and cells by reducing shear stress and providing fresh culture medium.

The establishment of an oxygen gradient to accurately reproduce oxygen zonation observed in the hepatic lobules was also achieved using a multilobule Liver-chip constructed by Ya et al.^153^ This model includes primary mouse HCs, LSECs, HSCs, and KCs, cocultured in an LLC which consists of superimposed PDMS chambers, inlets that resemble the portal vein and hepatic artery perfusion, and an outflow that resembles central vein perfusion, as shown in Figure 8a. LSECs exhibited self-assembly, resulting in the formation of a network with sinusoidal characteristics within the chip. Remarkably, this model represents one of the rare platforms which significantly improved physiological relevance by recapitulating multilobular architecture, zonation aspect, and complex vascular networks, furthermore enabled hepatotoxicity assessment and in situ tumor modeling.

**Figure 8:**
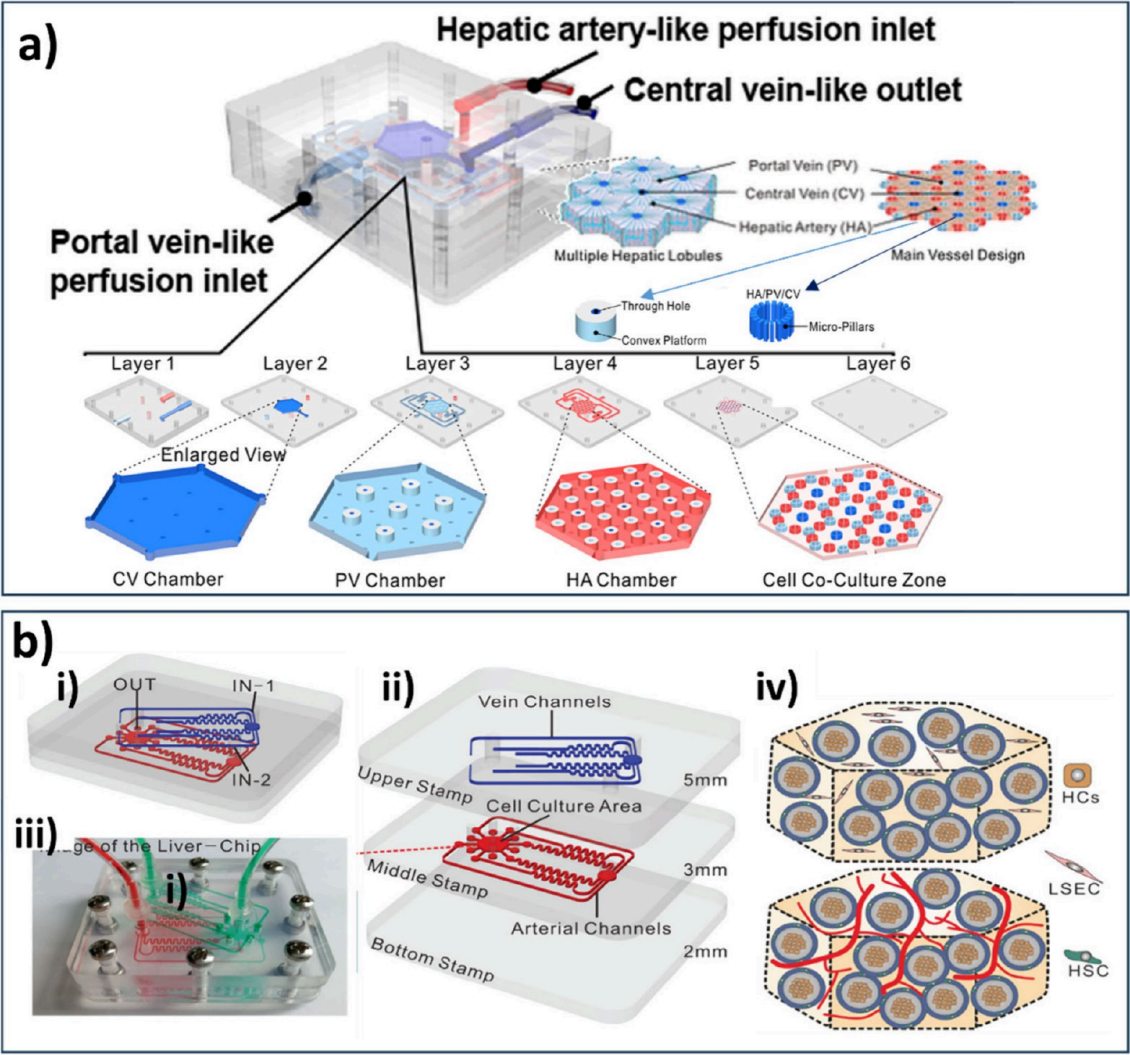
a) Conceptualization and development of a microfluidic multilobule LLC device, adapted with permission from ref 153. Copyright 2021 American Chemical Society. b) TVLOC system. Schematic depiction of (i) fully assembled TVLOC. (ii) various components of the TVLOC. (iii) Physical image of the assembled TVLOC. (iv) process involved in the formation of vascularized liver tissue within the culture area, reprinted from ref 108 and licensed under CC BY 4.0. Copyright 2022 Frontiers.

In another innovative approach, Liu et al.^108^ fabricated a trivascular liver-on-a-chip (TVLOC) that consisted of a hepatic artery fluid supply channel, a portal vein fluid supply channel, and a hepatic lobular hexagonal cell culture area (Figure 8b). Hepatic artery and portal vein cells were wrapped in bilayer microspheres generated through droplet-based encapsulation, while the hydrogel layer simulates the space of Disse. LSECs formed a vascular network around microspheres, creating vascularized liver microtissues in the culture chamber. The size of the microspheres should not exceed 400 μm, owing to to limitations in oxygen diffusion distance. This study found that the concentrations of metabolites (albumin and urea) increased over time, indicating the ability of TVLOC to sustain hepatocyte function effectively.

Alongside the cell patterning techniques used in the previously reviewed models, such as droplet-based, hydrodynamic, and microstructure-guided patterning, researchers have also explored the potential of laser patterning to create microchannels which enable the construction of continuous microvascular network (Figure 9a),^154^ and force-based patterning to manipulate both parenchymal and NPC arrangement. For example, Ho et al.^155^ fabricated hepatic lobule arrays for cocultivating HepG2 cells and HUVECs and achieving the desired pattern through dielectrophoretic force, which guides the cells to specific positions in a microfluidic chamber (Figure 9b). Remarkably, CYP450 enzymatic activity increased by 80% in patterned HepG2 cells compared to that in nonpatterned cells, emphasizing the importance of cell arrangement. Similarly, Chen et al. developed an LLC consisting of human hepatocytes (HepG2/C3A cells) and fibroblasts (NIH/3T3 cells) arranged in a radial pattern via dielectrophoresis (DEP) and hydrogel photopolymerization.^156^ Critically, such a patterning technique can enable precise spatial organization of different cell types within microfluidic chips, providing a powerful strategy for advancing LoC models. However, the surface properties of the electrode materials used in DEP, such as stiffness and hydrophilicity, may influence the phenotypic and functional attributes of both HCs and NPCs. Therefore, further research on 2D planar cultures should prioritize the development of novel suitable materials.

**Figure 9:**
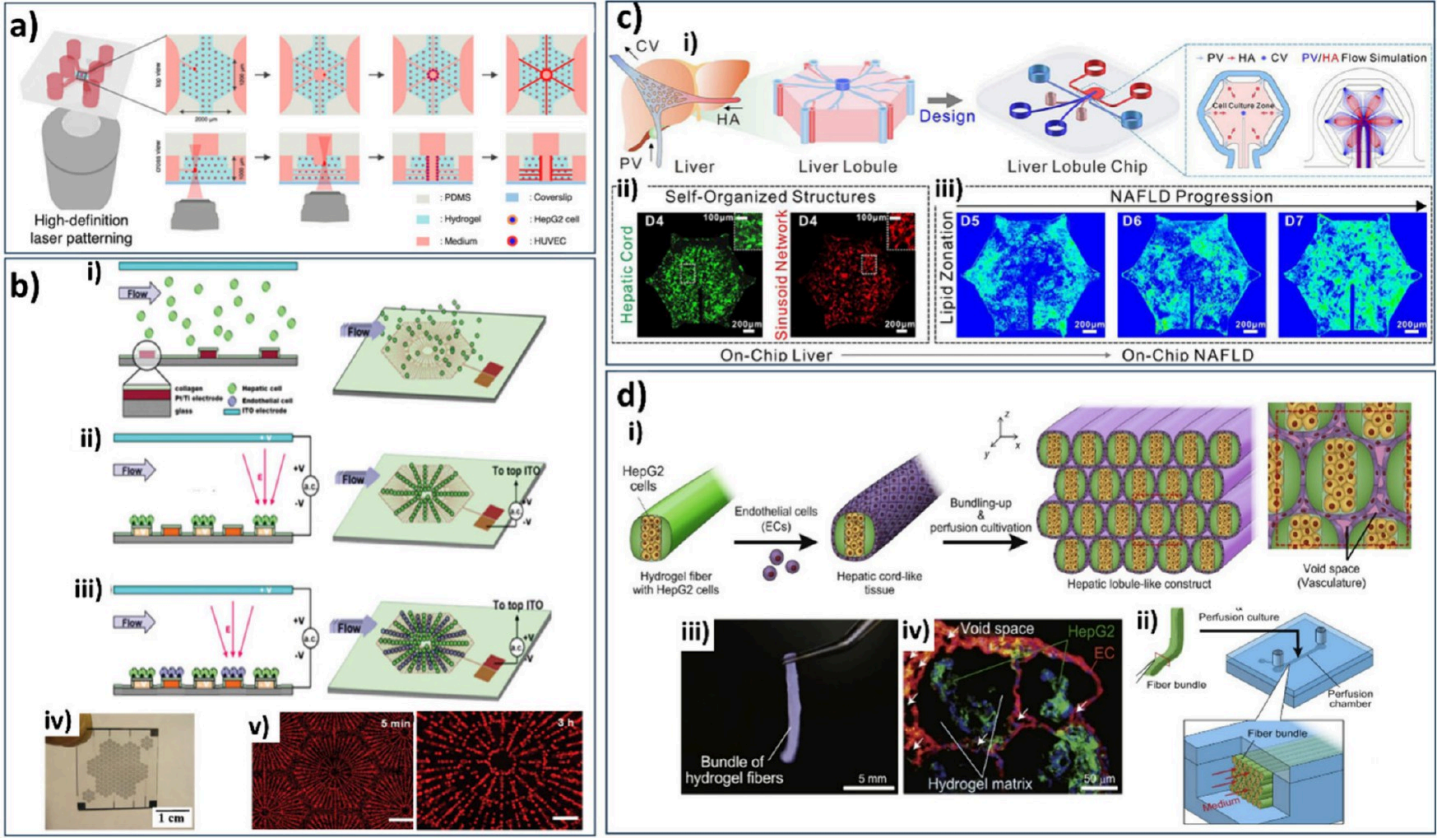
Conceptualization and development of different hepatic lobule chips. a) Fabrication process using high-definition (HD) laser patterning, reprinted from ref 154 and licensed under CC BY 4.0. Copyright 2025 Elsevier. b) Cell-patterning using DEP concept: (i) HCs put into the microfluidic chamber randomly and (ii) organized in a hexagonal arrangement via 1st DEP. (iii) ECs integrated between patterned HCs using 2nd DEP. Images of (iv) the chip and (v) the patterned cells (red-labeled cells: HepG2), reproduced from ref 155 with permission from the Royal Society of Chemistry. c) Construction of another—LLC. (i) The manufacturing methods used; an enlarged depiction of the LLC; and the simulated flow patterns inside the LC. (ii) Full view of the hepatic microtissue with cord-like and sinusoid-like structures on day 4 (HepaRG cells and HHSECs were, respectively prestained with DiO (green) and DiI (red) cell dyes). (iii) On-chip construction of NAFLD model: The distribution of lipids was assessed subsequent to incubation with a lipogenic media for durations through Nile Red intensity, reprinted from ref 157. Copyright 2021 Elsevier. d) Establishment of LLC using a bundling-up assembly technique: (i) Process of assembling cell-laden hydrogel microfibers and procedure of fabricating vascular network-like structures. (ii) Perfusion cultivation. (iii) Image of hydrogel bundle. (iv) Microfibers containing liver cells, adapted from ref 158. Copyright 2018 Elsevier.

In a recent study by Du et al.,^157^ an LLC consisting of three stacked PDMS layers with a dual blood supply was developed, featuring a culture chamber that mimicked the lobule size and structure, The cells were arranged via perfusion-driven self-organization (Figure 9c). Notably, this model has demonstrated improved performance compared to only a single blood supply. The chip achieved high cell viability, a self-organized hepatic cord-like structure, and a hepatic sinusoid-like network, enabling the development of a more reliable on-chip disease model. Moreover, this platform provided a bionic chemical microenvironment for inducing NAFLD (Figure 9c-iii). Importantly, the findings of this study could not be achieved by previously reported NAFLD models, which do not recapitulate the nutritional gradients associated with dual blood supply.

Yajima et al.^158^ introduced a new method for the perfusion culture of liver cells using cell-laden hydrogel microfibers. HepG2 cells were packed into sandwich-type anisotropic microfibers. The fibers were coated with vascular ECs and assembled together using perfusion culture to form conduit-like vascular networks (Figure 9d-i). The microfibers were then retrieved using a roller mechanism and subsequently consolidated at a single location to create a cohesive fiber bundle that was then placed in a perfusion chamber. Perfusion cultivation was conducted by injecting a medium for cell growth (Figure 9d-ii and (iii). Notably, this approach accurately replicates the structural characteristics of the hepatic lobule as observed in an *in vivo* setting, providing significant potential for various biomedical applications.

In conclusion, these LLC models have immense potential for advancing disease modeling, drug screening, and evaluation, particularly because of their enhanced ability to replicate the complex architecture and function of the liver. The vascularized system on these models accurately mimics the intricate network of blood vessels within lobules, enabling the study of tumor metastasis progression, as well as other critical research areas such as regenerative medicine and physiological drug testing.

### 3.3 Bioprinted Models

One of the difficulties encountered by LoC systems is that, despite HCs inherently have hundreds of important functions in the human body, most models, particularly 2D LoCs, can only recapitulate a few of those liver-specific functions.^159–161^ Extracellular matrix hydrogels are commonly used for tissue engineering,^162^ static 3D cell culture models^163^ and OoC models.^164^ For instance, a fibrin hydrogel-incorporated microfluidic chip used to culture HUVECs with MSCs allowed for the recapitulation of vasculogenesis and preservation of the endothelial barrier;^165^ nevertheless, the results were limited to 2 days. Therefore, for the transition from 2D to 3D cell culture and to achieve long culture periods, sophisticated methods, such as bioprinting, can be utilized. One of the earliest studies involved bioprinting HepG2 cells with alginate to create an LoC model.^166^ Currently, various techniques of bioprinting tissue-like constructs for 3D in vitro modeling have been reviewed, including strategies for hydrogel bioprinting and cross-linking.^167^

Integration of a bioprinted construct with microfluidic systems is required to enable perfusion in the system. To achieve this, a microbioreactor chamber is commonly used to house bioprinted constructs inside the LoC chip platform.^168^ GelMA is a hydrogel commonly used in such models,^169–173^ whereas HUVEC endothelial cells have frequently been preferred for bioprinting interconnected capillary-like networks to recapitulate angiogenic properties.^174–177^ Some studies are particularly promising because they overcome the poor mechanical properties of natural bioink materials, such as collagen gel and alginate, through the use of alternative polymeric materials, such as polycaprolactone (PCL), which offer better mechanical properties, allowing easier layer-by-layer printing.^174^ The mechanical properties of bioinks can be remarkably improved by appropriate selection of ECM materials, and the culture duration can be extended to 10 or 14 days.^174^ Such parameters are important when addressing the sustainability and reproducibility of LoC systems.

To compensate for the lack of both bioactive and mechanical properties of LoC endothelial-like barriers, decellularized extracellular matrix (dECM) obtained from liver tissue is a smart choice as a bioink material for bioprinting, furthermore, a variety of bioinks can be utilized in a single biofabrication process. In the study conducted by Lee et al.,^175^ HepaRG cells were incorporated into liver dECM bioink and bioprinted onto a microporous structure, followed by a second bioprinted layer that contained HUVECs in gelatin bioink. The chip design enables hepatic coculture on a platform with upper vascular and lower biliary fluidic channels. In addition to the advantages of bioprinting, the lower channel of this LoC model was beneficial, as it facilitated the removal of waste products and bile acids, promoting better biliary system creation and liver-specific functions. In a more recent bioprinting study, dECM was used with GelMA as a bioink in the digital light processing (DLP) technique for the biofabrication of liver microtissue, where the combination of dECM and GelMA yielded superior results in terms of growth, migration, and spreading of human-induced hepatocytes (hiHep cells) compared to GelMA alone.^169^

Another area of research has focused on mimicking the microarchitecture of the liver anatomical units through bioprinting. For instance, a recent extrusion-based bioprinting work attempted to imitate the lobule microstructure.^176^ HUVECs, human HSCs, and hepatocyte-like cells differentiated from adipose-derived MSCs were bioprinted in a bioink containing a mixture of porcine dECM, silk fibroin, gelatin, and β-D-galactose (Figure 10a). Another example is the work of Liu et al.^171^ who mimicked the blood vessels and bile ducts of the liver using bioprinted agarose microfibers covered with postprinted GelMA. After hydrogel cross-linking, the fibers were removed, and the hollow microchannels were filled with either RBE cholangiocarcinoma (CCA) cells or HUVECs to create a CCA-on-a-chip model (Figure 10b). To further construct a lobule-like complex structure, a DLP process was used to bioprint HepG2 cells, HUVECs, and RBE cells incorporated into their respective bioinks, which were photopolymerized using binary digital masks to form spatially complementary patterns of hepatic parenchyma, vessels, and bile ducts in interconnected hierarchical assemblies (Figure 10c). These advanced bioprinting methods provide new insights not only into the relationship between liver microarchitecture and function but also establish a promising path for developing more accurate LoC models, enabling enhanced drug testing and disease modeling.

**Figure 10:**
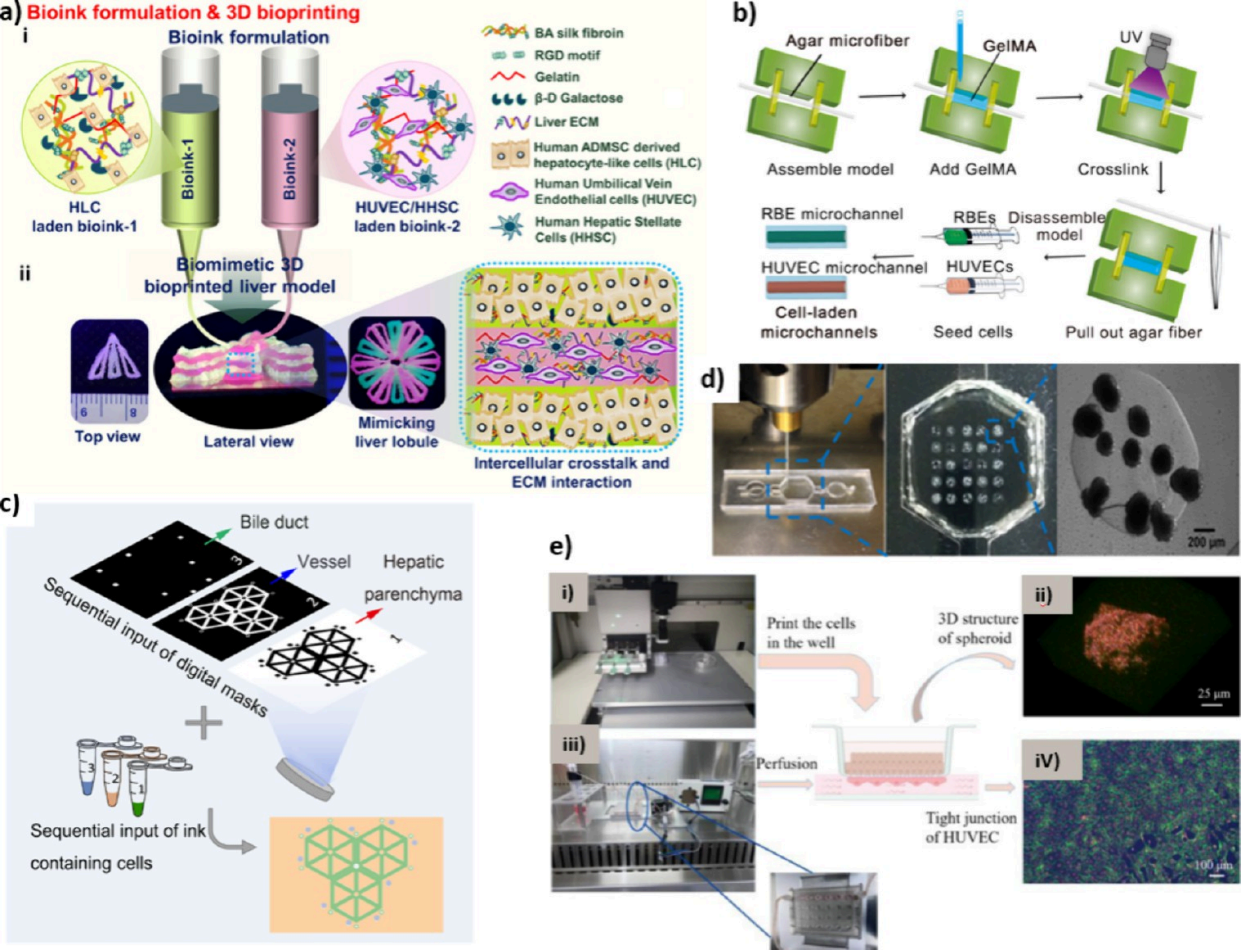
a) Schematic illustration of (i) bioink formulation and (ii) extrusion-based bioprinting for the lobule-like LoC model, reprinted with permission from ref 176. Copyright 2022 American Chemical Society. b) Production steps for GelMA microchannels via sacrificial bioprinting, followed by cell seeding. c) DLP bioprinting-based CCA-on-a-chip lobule-like model utilizing a three-step approach, b and c were reprinted with permission from ref 171. Copyright 2023 Springer Nature. d) Bioprinting GelMA-based hepatic spheroids in a dot array within an LoC bioreactor reprinted with permission from ref 172. Copyright 2016 IOP. e) Schematic diagram of bioprinted spheroids-on-a-chip: (i) HepG2 cell seeding on the upper side of the chip membrane by the bioprinter; (ii) image of the 3D spheroid structure captured by a confocal microscope; (iii) perfusion of HUVECs seeded on the bottom side of the chip membrane using a peristaltic pump; (iv) immunofluorescence image illustrating the tight junctions of HUVECs postperfusion, reprinted from ref 177. Copyright 2022 Elsevier.

Among the different 3D cell culture models, spheroids created by the aggregation of primary liver cells or cell lines compacted by the ECM have been the subject of several studies. Interestingly, Bhise et al.^172^ created tissue-like 3D constructs by bioprinting a HepG2/C3A spheroid-laden GelMA hydrogel as a dot array into a perfusable bioreactor (Figure 10d). This LoC model permitted a long culture period (30 days) and exhibited a response to APAP treatment similar to that in animal and in vitro models. Shin et al.^173^ encapsulated PHH spheroids in GelMA, followed by their bioprinting on a simple LoC platform integrated with electrochemical biosensors to monitor the secretion of albumin and GST-alpha. Such bioprinting approaches, together with integrated sensors, facilitate automated and high-throughput fabrication of advanced LoC devices. More recently, Tian et al.^177^ developed an LoC model featuring HepG2 spheroids, 3D-bioprinted on top of a transwell membrane precoated with collagen and Matrigel (Figure 10e). HUVECs were cultured on the bottom of the membrane with perfusion to simulate shear forces in vivo, effectively mimicking the hepatic sinusoid microenvironment and allowing an accurate assessment of hepatotoxicity, which was not possible with in vitro 2D and animal models. It is worth mentioning that combining bioprinting advantages with fluid perfusion on transwells offers synergical advantages, transforming simple transwells into scalable, sophisticated LoC models.

Although the previously mentioned bioprinting of spheroids or constructs allows for the fabrication of controlled 3D architectures and help overcome the low throughput—a typical problem encountered in liver MPS,^178^ they still encounter many challenges. Standardization and batch-to-batch variation are important issues related to the bioprinted models of LoC systems. Furthermore, bioprinting instruments require usually advanced engineering expertise, which may limit researchers having biological or medical backgrounds from fully understanding and employing these technologies. Addressing these challenges is essential for the adoption of bioprinting techniques in the fabrication of LoCs.

### 3.4 Advantages and Disadvantages of Complexity

Complex LoC models are powerful tools for performing drug metabolism and cell interaction studies due to their recapitulation of the complex liver microenvironment.^179–183^ These models not only mimic physiological blood but also combine different cell types in a 3D framework, essential for studying liver function and for implementation in more advanced applications. Although these innovative models represent a significant advancement, they also present complexities with both advantages and disadvantages.

#### 3.4.1 Advantages

As detailed in Section 3.2.2, some LoC models mimic both the complex architecture and the microenvironment of liver sinusoids, enabling improved hepatic functionality. Such complexity triggers many biological reactions, including the synthesis of urea, albumin, and vascular endothelial growth factors. Furthermore, such multicellular models enable the investigation of cell interactions, cytokine signaling, and paracrine effects.^182,184^

Some researchers have taken complexity a step further by creating models that aim to replicate not a single sinusoid but a larger liver structure containing multiple sinusoids arranged in a hexagonal pattern: the hepatic lobule (Figure 11). They not only simulated the spatial organization of HCs, as Banaeiyan et al. did,^119^ but also further incorporated nonparenchymal cells, as detailed in Section 3.2.3. For instance, Ho et al.^155^ have fabricated an innovative liver-lobule-chip (LLC) model (Figure 9b), where they emphasized how lobule mimicking facilitated the coordinated guidance, compression, and alignment of endothelial and hepatic cells. The importance of cell–cell interactions was particularly highlighted, and communication between cells was crucial for the success of the lobule-mimicking pattern, with hepatic cells showing high cell viability (∼95%) and an 80% improvement in CYP450–1A1 enzyme activity compared to monocultured, randomly distributed HepG2 cells. Thus, both cell arrangement and cell–cell interactions play key roles in cell viability, intercellular communication, and proliferation, as well as in the display of physiological and morphological properties of liver tissue that closely resemble the human in vivo environment.

**Figure 11:**
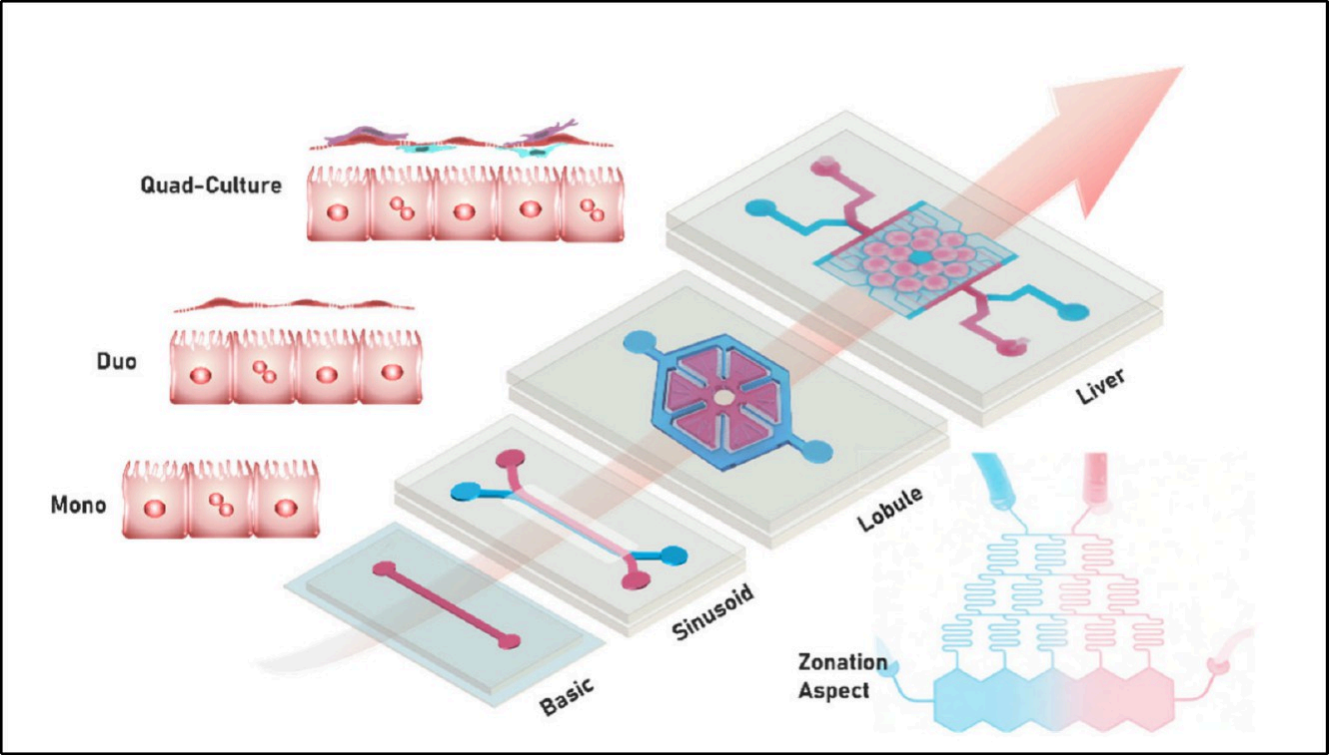
Schematic illustrating the different aspects of complexity levels in LoC models: from mono- to quad-culture, from basic to multilobule architecture, and the zonation aspect. HSCs and LSECs symbols were reproduced from NIAID NIH BioArt Source (bioart.niaid.nih.gov/bioart/565). The sinusoid-like chip design was reproduced from ref 109 with permission from the Royal Society of Chemistry. The lobule-like and multilobule-like chip designs were reproduced from ref 119. Copyright 2017 IOP. The design which explains the zonation aspect was reprinted from ref 116 and licensed under CC BY 4.0. Copyright 2018 Springer Nature.

In addition, some of these LLC models further replicate the functional differences between periportal and perivenous HCs by reproducing zonation,^152,153^ a crucial aspect for understanding metabolic processes and drug responses. Although some monoculture models exhibit this feature, as discussed in Section 3.2.1, the integration of zonation with coculturing and lobule architecture mimicking makes these models highly useful for evaluating therapeutics and predicting drug responses, thus advancing preclinical drug discovery.^107,185^ The researchers who developed the LLC models mentioned in Section 3.2.3 highlighted how complexity in their models, such as integrating several mechanical forces, zonation aspect, and different types of cells, enables more precise assessments of drug toxicity and immune reactions, which would not be achievable in less complicated models. They also elucidated how this complexity also helps develop more accurate disease models, such as hepatitis, NAFLD, ALD, and DILI.^16,179^

Finally, a key advantage of complex LoC models is their ability to integrate HCs differentiated from human-induced pluripotent stem cells (hiPSCs) to facilitate in situ differentiation, enabling patient-specific liver models for tailored treatments. Current designs include sinusoid-like^186^ and lobule-like^119^ structures as well as three-channel configurations.^187^ Some models feature microfluidic chambers with micropillars^188^ or shaped traps^189^ to confine cell aggregates, particularly for 3D liver organoids. Usually, specific hydrogels are used to encapsulate cells, ensuring stable hepatic function. While many models employ coculture systems,^186,187,189^ only Suominen et al.^187^ have compared coculture with monoculture, demonstrating that the multilineage LoC model significantly outperformed monocultures. This again highlights the importance of cellular interactions in maintaining liver functionality, while promoting personalized medicine. These LoC models have the potential to enable the customization of certain medicines according to the patient’s genetic composition and disease features, which could significantly advance precision medicine.^190^

#### 3.4.2 Disadvantages

Although complex LoC models show immense potential, they also have several serious drawbacks.

Despite possessing biorelevant geometries, complex LoC models, such as lobule-inspired models, have complex fluidic networks, multichambered layouts, micropatterned structures, and elaborate shear stress profiles. Although simulation studies have been conducted prior to most experimental studies on these models, a deeper understanding of the physics governing their complex microfluidics and mass transport, particularly in the presence of different cell types, is still required.

Moreover, these complex devices are usually manufactured and implemented using technologically advanced processes such as photolithography and dielectrophoresis, as discussed in Sections 3.2.2 and 3.2.3. These techniques add complexity, time, and resources to the process^179,191^ Furthermore, they increase the budget required for the project and the level of technical competence needed to operate their tools. As stated in Section 3.3, bioprinting offers a promising approach that enables the precise spatial arrangement of cells to replicate the liver structure.^192–194^ . However, elaborate designs require sophisticated equipment and technical expertise in both 3D printing and cell culturing. Because of these complications, these models are still considered experimental tools rather than standard diagnostic tools; nonetheless, further research attempts to lower costs and streamline fabrication methods, in order to make these systems more widely available.

Another issue resulting from the complexity of many LoC platforms is their high maintenance and optimization costs.^6^ The intricacy of building functional liver systems poses many difficulties. Any lack of consistent reproduction of lobule-like structures, intertissue interactions, and trivascular systems render their maintenance challenging, thereby negatively impacting cell survival and differentiation.

An expected drawback of all LoC models is the lack of complete physiological representation. Even the very sophisticated ones are still insufficient to accurately reflect the complex microenvironment of the liver and cannot fully account for all the dynamic interactions that occur in the native tissue. For example, the bile duct and its cholangiocyte cells are often overlooked and mimicking the zonation feature to maintain an oxygen gradient, which is critical for metabolism, remains a challenge in many complex LoCs.

In addition to these challenges, the large-scale adoption and commercialization of complex LoC prototypes may be hindered by other factors; although more realistic functionality can be provided by more complicated models, it is very important to consider the raised reproducibility, scalability, and long-term stability issues. Moreover, the interpretation of results derived from scientific studies that involve complex LoC models and their extrapolation to clinical settings maybe very difficult. In fact, the use of multiple cell types with distinct roles and behaviors complicates analysis of data and identification of meaningful patterns; the integration of different data types creates difficulties in data visualization and interpretation.^195^ Therefore, collaboration among scientists, engineers, and computer scientists is essential for realizing the full potential of complex LoC models for drug development and medical research.

## 4 Case Studies of LOC Commercial Models

The Wyss Institute at Harvard University has been at the forefront of developing innovative OoC technologies, including LoC models that were later commercialized by Emulate Inc.^196^

Similar to Emulate, Inc., different companies active in the field of OoC have begun to fabricate various LoC models over the past decade. These models are commercially available and used by different research groups and industrial users. Below is a list of companies that produce LoC platforms with information gathered from their respective Web sites.Table 2 provides other details regarding their manufactured chips, such as design images, microfluidic materials, and pumping systems.

**Table 2:**
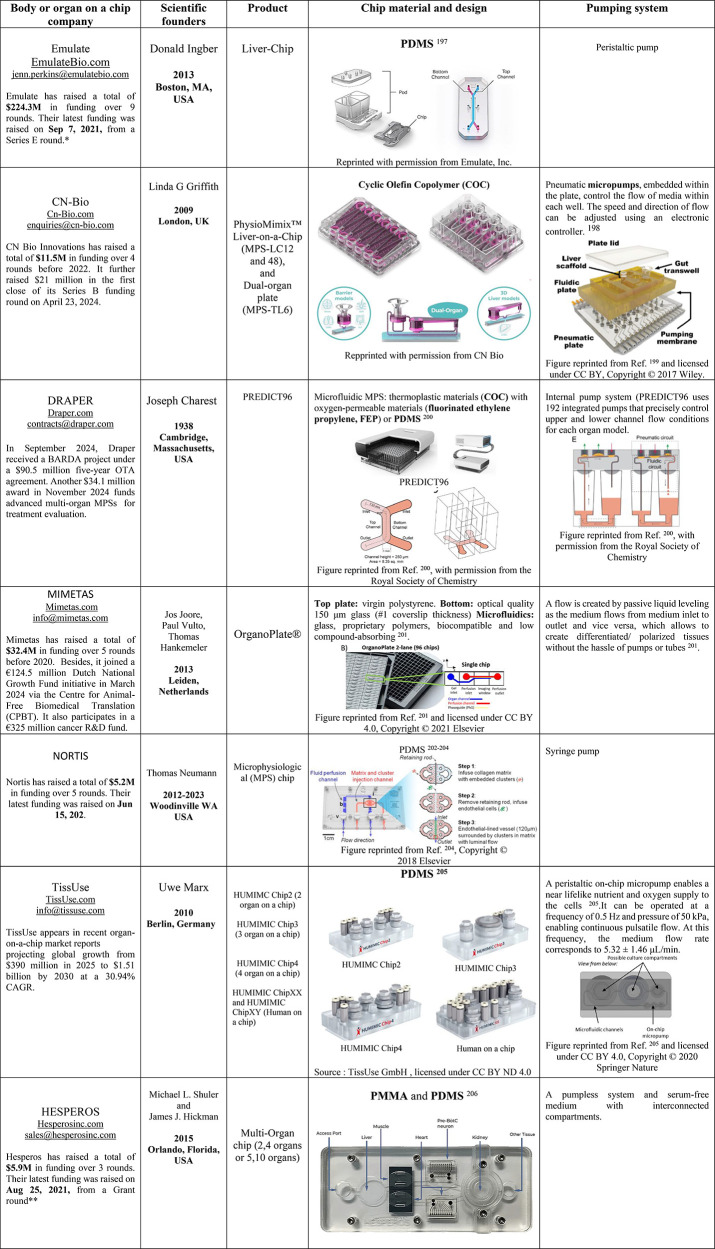
Commercial Liver on a Chip Models

### 4.1 Emulate

The Emulate Human Liver-Chip represents a groundbreaking advancement in liver modeling by incorporating up to four distinct human cell types within a dynamic microenvironment.

The chip replicated the sinusoidal architecture and consisted of two overlapping channels with a membrane in between. The upper channel is the epithelial channel, where PHH (without stellate cells) can be seeded on the membrane. The lower channel is the endothelial channel, where human LSECs (w/wo Kupffer cells) are seeded on the opposite side of the membrane. This arrangement can handle both coculture and quad-culture configurations depending on the study requirements.

Jang, K.-J. et al. investigated the species-specific capacity of Emulate’s Liver-Chips of predicting DILI using rats, dogs, or human PHH and NPCs. The human liver chip produced comparable levels of albumin to human in vivo liver and higher cytochrome P450 enzyme activity than traditional monoculture plates. When treated with bosentan drug, all species chips showed reduced albumin secretion, but only the human and dog liver chips matched clinically relevant toxic responses, demonstrating species-specific sensitivity. The study highlighted the ability of EMULATE chips to model DILI mechanisms over conventional models.^197^

### 4.2 CN-Bio

CN-Bio is a biotechnology company that focuses on the development of OoC technology. CN-Bio liver models are divided into two categories: single and multi OoC models.

- **Single organ model:** This LoC model is designed for studying metabolism, toxicology, and liver diseases. It consists of 12/48 independent bioreactors integrated into a single plate with a recirculating perfusion system to stimulate the blood flow. Each reactor contains a collagen-coated scaffold featuring microchannels to promote cell adhesion and 3D microtissue formation, enabling long-term studies. This platform is provided by CN-Bio in either mono, duo, or triculture configurations, with the possibility of incorporating additional cell types depending on the targeted application. Moreover, it offers other advantages, including the ability to conduct multiple experiments in triplicate on a single plate with high inter- and intraplate reproducibility, permitting the obtention of robust and reliable data. Additionally, the built-in micropumps and flow controllers of the system ensured an optimal cell environment, supporting long-term viability and accurate results.
- **Multiorgan-on-a-chip:**The accurate prediction of ADMET in preclinical trials by modeling -not only one- but also multiple organs is crucial for simulating their functions as well as for determining any side effects of the pharmaceuticals.^207^ In this context, multi-OoC systems that incorporate human-derived cells hold significant promise. Additionally, increasing knowledge about the relationship between dysfunction in metabolic crosstalk and chronic diseases, such as type 2 diabetes and NAFLD, has increased the importance of multi-OoCs.^208^ These systems enable the modeling of interactions between metabolically active organs, including liver, gut, and pancreas, in a human-specific context. Consequently, various multi-OoC models have been developed and effectively applied in drug development and disease modeling.
  - **Gut/Liver-on-a-chip model:** CN-Bio has interconnected the previously mentioned liver-chip model with the gut-chip model via fluidic flow to explore organ–organ crosstalk, predict toxicity, and model inflammatory disease processes. This model allows for the examination of the combined effects of intestinal and liver metabolism, facilitating the comparison of oral and intravenous dosing treatments for evaluating drug bioavailability. Its open system configuration enables the sampling of circulating drugs, metabolites, and biomarkers for concentration–time profile generation. Chen et al. used this model with primary cells to simulate gut-liver interactions over 2 weeks, maintaining key liver and gut functions, including albumin production and barrier integrity. This study confirmed the complex communication between the gut and liver during inflammation, supporting the model’s potential for drug discovery and pathophysiological studies.^198,199,202,203,204^

  - **Lung/Liver-on-a-chip model:** This model is intended to predict drug safety, efficacy, and metabolic pathways; it helps determine the PK of inhaled or intravenously administered drugs and compare their effects in both healthy and diseased models. This platform sheds light on the interactions with circulating immune cells, generating an inflammatory response to stimuli administered in either the lung-chip or liver-chip.

### 4.3 Draper

The Draper company has significantly contributed to the advancement of OoC technology. Its PREDICT96 system has 96 independent OoC units implanted in a conventional 96-well plate format, designed for easy integration with standard laboratory equipment and pharmaceutical pipelines. The flexibility and high throughput of this platform permit achieving 5 to 10-fold the data output obtained using other platforms. The design of OoC units consists of two superimposed microfluidic channels separated by a Transwell-like membrane. Interestingly, this platform controls perfusion using 192 independent micropumps, which allows for precise control of media volume and seeded cell density. Furthermore, the incorporation of parallel-integrated sensors enables more predictive preclinical testing for drug development and opens new avenues for novel drug discovery.

Tan et al.^200^ used this system, in which HCs were cultured in a collagen sandwich configuration, with media exchange facilitated by a second channel beneath the cell culture channel. The ability of the platform to maintain unidirectional fluid flow around the 3D hepatocyte culture helps mimic the in vivo liver sinusoidal blood flow. Assessment of hepatocyte function, including albumin secretion and Cytochrome CYP3A4 enzymatic activity, showed that dynamic flow conditions significantly enhanced hepatocyte function, as proven by increased levels of albumin, α1-antitrypsin, VEGF, and HGF, as well as by achieving sustained CYP3A4 activity until day 13, in contrast to the declining activity in static cultures. Therefore, the PREDICT-96 platform efficiently models human liver functions, providing an advanced tool for drug metabolism and hepatotoxicity studies.^209^

### 4.4 MIMETAS

The specific feature of MIMETAS multichip plates is the ability to perform trans-epithelial/endothelial electrical resistance (TEER) and transport measurements, which enables real-time monitoring of barrier integrity and cellular activity.

MIMETAS microfluidic platforms, known as OrganoPlate family, were designed specifically for 3D tissue cultivation, which involves embedding cells in a gel matrix to mimic the operation of human organs. They have two-lane, three-lane, or graft configurations that accommodate different coculture arrangements. Barrier models function best with a 2-lane layout, whereas 3-lane formats support more intricate systems such as vascularization. The platform is available in 96-, 40-, and 64-well configurations, enabling experimental scalability. The OrganoPlate perfusion system is a crucial component that ensures that cells receive adequate amounts of nutrients and oxygen by imitating the blood flow through microchannels. Notably, this system does not require external pumps because of its gravity-driven flow. Furthermore, OrganoPlate offers basolateral and apical access, which promotes precise drug absorption and permeability experiments, and facilitates the investigation of chemical transport across barriers. Finally, OrganoPlate offers flexible and physiologically realistic technology for modeling human tissues and organs, including the liver. Furthermore, the automation compatibility makes these models perfect for high-throughput drug screening using automated liquid-handling systems.

### 4.5 Nortis

Nortis company developed its own chip named ParVivo, which was the world’s first preseeded chip system that allows researchers to use functional living human tissues embedded with chips without needing to engineer the tissues themselves.^210^ This ″plug-and-play″ model simplifies the research processes by providing pre-engineered, high-quality tissues manufactured by Nortis under strict quality control, minimizing variability among chips. This approach was later followed by companies such as Emulate and CN-Bio, which now offer validated and complete solution kits. The Nortis OoC system was based on a microfluidic platform with dedicated channels for (i) extracellular matrix (ECM) injection and (ii) fluid perfusion. The fluid perfusion channel controls directional media flow through a cylindrical lumen lined with endothelial cells, while the second channel contains a 3D scaffold by injecting and polymerizing collagen solution. To further control the flow and provide precise experimental conditions, a bubble trap to stop air bubbles from obstructing the flow and a retaining rod for creating a lumen in the collagen matrix are also integrated. After the matrix polymerizes, the rod is removed, revealing an endothelial cell-lined hollow channel that allows the medium to pass through the specially designed vessel.

Chang et al.^211^ used the Nortis LoC model to evaluate hepatotoxicity and to predict drug safety and efficacy. These findings indicate that LoC-cultured human hepatocytes remained viable for up to 15 days, with minimal viability in 2D cultures at day 8. Nortis LoCs demonstrated prolonged and improved hepatic function, including enhanced albumin production, HNF4α expression, and inducible CYP activities, when compared to 2D cultures. Furthermore, it effectively assessed the acute toxicity of aflatoxin B1, showing higher accuracy than conventional 2D cultures.^211^

### 4.6 TissUse

Diverse range of HUMIMIC Chips are offered by TissUse as follows.

- **HUMIMIC Chip2:** Designed to sustain 3D cultures such as spheroids or matrix-supported tissue models such as the liver, as well as biological barriers such as the colon, lungs, or skin. These organ models are connected via microfluidic channels, and an on-chip pump mechanism which ensures that the cells receive oxygen and nutrients constantly and realistically. The chip structure consists of various crucial parts. Transparent and flexible PDMS constitutes the microfluidic layer, and the adaptor plate is composed of clear polycarbonate. To comply with ISO standards, a tiny glass slide was employed, and PEEK and polycarbonate were used to construct the cell culture compartments, which provide the optimum conditions for cell culture. Three 500 μm-thick pump membranes are part of the pump mechanism integrated within the chip. These membranes function similarly to the biological fluid flow by employing vacuum and air pressure. Designed to be flexible for research purposes, HUMIMIC Chip2 supports the delivery of substances in two ways, apically (top layer) and systemically (medium-based). A control unit such as the HUMIMIC Starter is necessary for the HUMIMIC Chip2 to operate fully, which makes it an extremely flexible and sophisticated instrument for simulating physiological conditions and multiorgan in vitro models.
- **HUMIMIC Chip3 and HUMIMIC Chip3plus:** Building upon the advantages of HUMIMIC Chip2, both HUMIMIC Chip3 and HUMIMIC Chip3plus provide the same features while accommodating an additional organ model.
- **HUMIMIC Chip4:** facilitates the easy integration of up to four different organ models, such as a combination of intestine, liver, kidney, and neuronal tissues, in one system. The PBPK-compliant design enables quantitative *in vitro* and *in vivo* extrapolation. Using two separate microfluidic circuits, the HUMIMIC Chip4 can model different processes such as excretion and reabsorption in the kidney model.

Schimek et al.^205^ reported a human multiorgan chip model for the coculture of bronchial lung cells and liver spheroids. The study utilized the HUMIMIC Chip3plus, which features a three-compartment system for optimal nutrient supply and oxygenation. This model demonstrated remarkable performance over a 14-day experimental period. Bronchial MuciAir tissues exposed to Aflatoxin B_1_ (AFB1) exhibited a drop in viability and functionality when cultured alone. Contrarily, coculturing with liver spheroids showed a protective effect against AFB1 toxicity, highlighting effective lung-liver crosstalk within the chip platform. The findings of this research hold promise for high prediction of human responses to inhaled substances and contribute to the development of safer pharmaceuticals.

### 4.7 Hesperos

This company manufactures physiologically relevant multiorgan models by including interconnected organ compartments such as heart, liver, lung, brain, e etc.^212^ These models operate without the need for pumps and utilize a carefully designed gravity flow system. Hesperos’ models, examining responses to various treatments by reproducing functional aspects of specific disease states, provide the basis for safety and efficacy analyses^212^ and examine effects on drug–drug interactions, ranging from single to multidrug treatments. The multiorgan chip models that include the liver produced by this company are as follows:

- Two-organ heart-liver model: ideal for investigating the efficacy and safety of new chemicals. This model combines complex functions, such as cardiomyocyte (CM) contractile force, beat frequency, and field potential duration.^213^
- Three-organ model: suitable for the safety of therapeutic index determination with heart-liver-cancer integration. It demonstrates drug efficacy, safety, and metabolite formation on multidrug-resistant (MDR) and non-MDR cancer cells.
- Four-organ model (Heart-Liver-Neuron-Skeletal Muscle): characterizes basic physiology in detail using MEA and console-based functional readouts. This model noninvasively monitors CM contractile force, skeletal muscle force, and neuron spontaneous action potential.^212,214^

- Heart-liver-skin model: evaluates the safety and effectiveness of topically applied products.

McAleer et al. used an in vitro heart-liver Hesperos model with hiPSC-CMs and primary human hepatocytes to study the pharmacokinetic-pharmacodynamic relationships of drugs over time.^206^ This system successfully detected terfenadine-induced QT prolongation in heart cells and its metabolism to fexofenadine, which lacks this cardiotoxic effect. This study demonstrates the importance of incorporating liver metabolism into the system and highlights its potential for enhancing early drug discovery and cardiac safety assessments.^206^ Kostrzewski et al. developed a fully human in vitro model based on the Hesperos OoC platform, in order to closely replicate the response of human body to NASH disease.^215^ PHH, KCs, and HSCs were tricultured under specific conditions which induced steatosis that progressed to NASH. The established model is considered a promising tool for understanding liver disease at the cellular level for more reliable drug discovery, providing a robust alternative to conventional preclinical rodent models.^215^

## 5 Discussion

### 5.1 Comparison between Academic and Commercial Models

After detailing academic LoC models reported in the literature as well as commercial models described on the official Web sites of their manufacturing companies and in some scientific papers, we will conduct a systematic comparison between them in this section. While some companies such as Emulate have successfully transitioned their LoC technology from lab to fab, the broader landscape of academic and commercial LoC models exhibits wide-ranging similarities and differences in terms of materials, complexity, and the ability to integrate into multiorgan systems (Figure 12). The examination of these factors will help scientists and industry users select the model that effectively aligns with their requirements. This comparison is also important, since it highlights the direction of future developments in LoC technology and guides further advancements.

**Figure 12:**
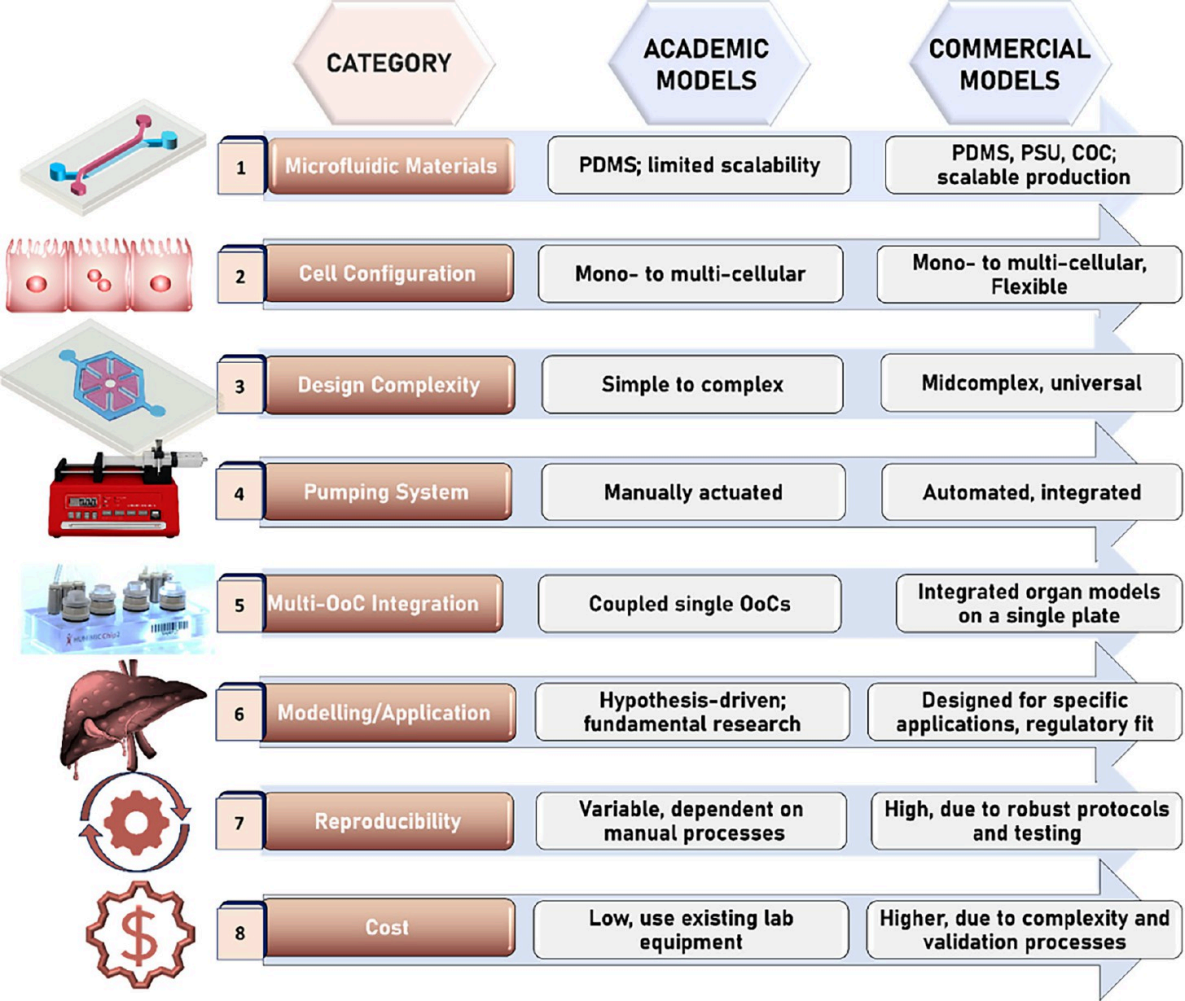
Academic vs commercial LoC models. The 1st symbol was reproduced from ref 109 with permission from the Royal Society of Chemistry. The 3rd symbol was reproduced from ref 119. Copyright 2017 IOP. The 4th and 7th symbols were AI-generated using ChatGPT based on descriptive input.

#### 5.1.1 Microfluidic Materials

PDMS is the primary material used for constructing microfluidic systems in both academic and most commercial LoC models (e.g., EMULATE, Nortis, TissUse, and HESPEROS). Its widespread use is mainly owing to its high gas permeability, which is approximately 3 orders of magnitude higher than that of PMMA and Cyclic Olefin Copolymer (COC).^101^ When a material with low gas permeability is used, oxygen for cell culture must be supplied through the medium circulating in the chip, which complicates the setup. Additionally, the cell-to-medium volume ratio is lower than that in a culture flask, thus requiring sufficient medium flow to prevent O_2_ deficiency.^216^ However, this can result in high shear stress, which many cells, particularly PHH, cannot tolerate owing to their high sensitivity to shear stress while having a high oxygen uptake rate.^217,218^ In this context, PDMS has a major advantage, as its permeability enables oxygen uptake from the ambient air outside the cell culture chambers.^219^

The substrate stiffness also plays a key role in LoC systems. PHH cultured on soft (2 kPa) PDMS maintained their functional, differentiated phenotype longer than those on stiffer (55 kPa) PDMS, whose Young’s modulus was adjusted by varying the polymer base-to-cross-linker ratio.^220^ Hepatic stellate cells are sensitive to surface stiffness.^221^ However, PDMS has limitations, covered in the “Challenges” section, including limited scalability due to soft lithography. Thus, companies such as CN-Bio and Draper use thermoplastics such as COC, respectively, because they are more easily mass-produced through micromachining, injection molding, or hot embossing.

#### 5.1.2 Cell Configuration

There is no standard approach for the number and types of cells in LoC models reported in academic literature. As discussed in Sections 3.1 and 3.2, these models vary from monocellular to multicellular systems. The advantages and disadvantages of this complexity are discussed in Section 3.4. Commercial models also reflect such diversity, with some companies utilizing HCs alone, especially in their multiorgan chips, as in the case of Hesperos. Other companies have employed coculture systems containing up to four cell types that develop complex cellular interactions and signaling. However, most commercial LoC platforms are designed to be flexible, allowing them to be tailored for particular needs. For example, CN-Bio offers three models: a monoculture of PHH for studies on drug metabolism, a duo-culture with KCs for inflammation studies, and a triculture with KCs and HSCs for NASH modeling.^222,223^ The approach aims to find a compromise between standardization and customization in order to make the technology widely available while supporting more advanced applications. Novel drug development can significantly benefit from minor increases in physiological relevance as hepatic cells exhibit more in vivo-like characteristics when physiological cues are restored. Meanwhile, high physiological mimicry in LoC devices is valuable for disease modeling, aiding in the research of disease onset, progression, and treatment.^101^ However, the integration of more complex combinations of NPCs still requires further optimization, and the advantages and reproducibility of these advanced models remain to be confirmed^224^

#### 5.1.3 Design Complexity

There are no set rules for academic LoC models. Some researchers have demonstrated that certain phenomena can be studied with simple designs, such as a monomicrofluidic channel,^113^ while others strived to mimic the liver’s complicated microscopic architecture of the lobule (Section 3.2.3). Others have focused on the level of sinusoids with basically a small, two-channel design divided by a membrane. This design is also common in commercial LoCs, such as Emulate and PREDICT96, owing to its relative simplicity and ability to mimic physiological barriers in several organs, including the lung, gut, and brain, making it universal for many OoC models^225,226^

#### 5.1.4 Pumping System

Most of academic LoC models, as well as some commercial ones, employ simple external pumping systems that are manually actuated, such as syringes or peristaltic pumps. These are preferred because of their low cost and ease of integration with novel chips/models, as academia often focuses on exploring new concepts and prototyping systems, rather than producing polished products. In contrast, most companies opt for integrated, automated pumping systems for improved ease of use and precision (e.g., CN-Bio, PREDICT, TissUse). However, some companies (e.g., Mimetas and Hesperos) and a few researchers^117,227,228^ have chosen gravity-driven flow to eliminate the need for pumps, electrical power supplies, and tubing, offering simplicity and cost-effectiveness

#### 5.1.5 Integration with Multi-OoC

Many studies have reported the use of custom-built multi-OoC systems that include LoCs, as summarized in Deng et al. review.^229^ Recently, some researchers developed their own chips.^227,230–233^ Others prefer commercially available chips for their studies,^234–236^ possibly due to complexity issues. While academic multi-OoC systems are typically coupled with single OoCs, commercial systems often integrate various organ models on a single plate (e.g., TissUse and HESPEROS), aligning more closely with the body-on-a-chip philosophy

#### 5.1.6 Modeling/Application

Both academic and commercial models are used for various applications, including disease modeling,^16^ drug screening,^107^ and toxicity assessments.^1^ However, academic models address specific hypotheses and research requirements that can extend beyond the frontier of current knowledge, to enable fundamental research and new discoveries. On the other hand, commercial LoCs are fitted for specific applications with a focus on industrial standards. They have generally developed application protocols for drug toxicity studies and are therefore well suited for regulatory submissions

#### 5.1.7 Reproducibility

One of the most crucial factors in determining the reliability of OoC systems, including LoCs, is reproducibility, which may be difficult in academic models owing to manual preparation, low control of operating conditions, and variability in chip materials, extracellular matrices, and cells. This may explain the lack of studies assessing the reproducibility of the LoCs produced by individual laboratories. In contrast, companies aim to produce robust and reliable models, and many offer multichip plate configurations (e.g., CN-Bio, PREDICT96, MIMETAS) that ensure, among other advantages, uniform conditions across all LoC units, thereby improving result reproducibility—an essential aspect for industrial and regulatory acceptance. Commercial manufacturers benefit from well-established protocols and invest in extensive testing to prove their reliability, thereby validating their platforms.^237^ The largest OoC study to date using a cohort of 870 LoC devices from Emulate was tested for the toxicity risk of 27 known drugs.^6^ The data showed high sensitivity (87%) and specificity (100%) across experiments, proving the reproducibility of the model, which makes it highly attractive to academic and industrial users

#### 5.1.8 Cost

Even within the same category, the cost of academic and commercial LoC models will be very different due to several reasons such as materials, complexity in design, and microenvironment. In general, academic LoCs tend to have much lower initial costs because most researchers utilize existing equipment from their laboratories and low-cost materials, such as manually cast, punched, and assembled PDMS. They also opt for manual cell seeding, which further lowers the overall cost despite being more labor-intensive. In academia, funding for research usually arises from grants, which may limit the scale and scope of projects to focus on innovation and proof-of-concept studies with a lack of standardization, which is much considered in commercial systems. For their part, commercially available LoCs are usually costlier because of the sophisticated systems deployed, scalability and validation processes, an optimized, reproducible, and ready-to-use platforms.

### 5.2 Challenges and Prospects

Both academic and commercial LoC models face several key challenges while offering promising prospects as this technology continues to evolve, many of which are shared across other OoC systems, as detailed in this section and illustrated in Figure 13.

**Figure 13:**
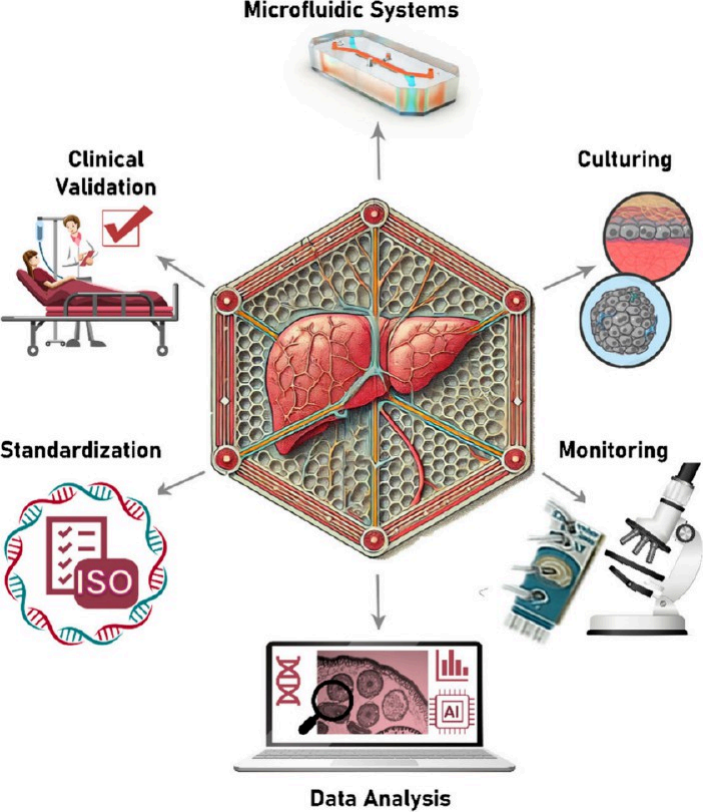
Schematic illustrating challenges and perspectives of LoC models. The cell culturing symbols were reproduced from the TOC of ref 273. Copyright 2016 ACS. The symbols of microscope, computer, circular DNA helix, doctor and patient were reprinted from Vecteezy.com. The screen-printed electrode was reprinted from ref 274 licensed under CC BY 4.0. Copyright 2025 IOP. The central schematic was AI-generated using ChatGPT based on descriptive input.

#### 5.2.1 Standardization

Despite the huge advancements made in diverse design and fabrication methods of LoCs, key challenges such as standardization, automation, scalability, and reproducibility still persist. This diversity mirrors the early development of microchips, where various designs emerged before standardization became more universal. Achieving conformity across both laboratory and commercial models is made more challenging by variations in cell sources and experimental conditions, limiting broader adoption. Automation is still not widely integrated, which affects accessibility and scalability, particularly for high throughput applications. The variety of design complexity levels, from very simple to highly complex or even multiorgan systems, adds another layer of difficulty to any attempt at establishing a single, unified standard, since each design serves different experimental goals. Even well-established commercial LoC models have not yet received clinical application approval, despite being actively evaluated by the FDA over the past few years,^237,238^ primarily due to the lack of standardized protocols for performance testing and validation

However, many efforts are being made; for instance, at the MPS world summits, this topic continue to be one of the key focuses.^239^ The ISO 22916 standard for microfluidic systems is a promising example that aims to improve consistency and reliability across different systems, providing a pathway toward industrial adoption.^240^ Additionally, collaborative efforts, such as the IQ-MPS consortium, 3RsC and EUROoCS, which bring various stakeholders together from academia, industry, and regulatory agencies, are actively working on establishing the best guidelines for a standardization roadmap.^241,242^

#### 5.2.2 Microfluidic Systems

The chip material plays a crucial role in maintaining the functionality and reliability of the model. As noted in Section 5.1, PDMS is the most commonly used microfluidic material in both academic and commercial systems owing to its biocompatibility, transparency, and ease of fabrication, usually achieved by soft lithography.^101^ Furthermore, it allows for the creation of complex and versatile microfluidic structures. However, PDMS’s tendency to absorb small lipophilic molecules can interfere with accurate drug metabolism and toxicity assessment.^243^ This absorption can cause false negatives in pharmacokinetic (PK) and ADMET profiling because it reduces the effective drug concentration, making PDMS less suitable for certain drug evaluations. Additionally, its permeability may permit culture media evaporation, leading to instabilities in long-term culture studies.^244^ Modification of PDMS by either surface treatment or impermeable coatings has helped reduce the absorption of small molecules and improve stability in drug metabolism studies. Hybrid PDMS materials were also developed to enhance chemical resistance while maintaining flexibility and ease of fabrication.^245^ As an alternative, thermoplastics such as cyclic olefin copolymers (COC) have gained attention; unlike PDMS, COC is more chemically resistant and less prone to nonspecific binding, making it better for drug metabolism and toxicity studies. Furthermore, its low gas permeability reduces evaporation in long-term cultures.^246^ Besides, the fabrication of COC-based microfluidics offers a much higher scalability, as emphasized in Section 5.1.1. However, COC has its own limitations; COC’s low gas permeability hinders oxygen exchange, which is crucial for cell cultures, and its high stiffness limits the ability to obtain complex microfluidic designs^14^

Another issue for many LoC models is the necessity of chip dismantlement to perform end point analyses such as histology, transcriptomics, lytic assays, and cell viability assays. Consequently, the irreversible sealing using PDMS plasma treatment has often been replaced by reversible approaches, including mechanical, magnetic, and gecko-inspired clamping, each of which has its pros and cons.^247^

Declogging from air bubbles and optimization of fluid flow within the microfluidic channels is also challenging.^248,249^ OoC, including LoC devices, may fail due to the lack of nutrients and oxygen at low flow rates. To address this issue, increasing either the radius of the flow or the pressure is necessary. Several commercial companies such as Emulate, CN Bio, and TissUse, have favored the latter approach, which inevitably increases the capital costs and control system complexity. In contrast, the first approach of scaling chambers and tubing diameters up to milli-fluidic levels was adopted by Kirkstall, for example, where the optimal flow can then be adequately provided at lower pressures but requires the use of peristaltic pumps instead of pneumatic pumps.

Another important topic to point out is the use of recirculating versus single-pass flow; recirculation promotes the continuous exchange of signaling molecules and metabolites, ensuring chemical communication between different model compartments. However, the lack of continuous replenishment of nutrients and the buildup of waste products may be detrimental.^248^ Thus, optimizing the duration of recirculation and the amount of medium used for refreshment in an OoC microfluidic system is essential. In this context, computer simulations are particularly valuable.

#### 5.2.3 Sensor Integration

Sensors offer a noninvasive method to monitor critical parameters such as oxygen levels, pH, glucose, and lactate, which is essential for maintaining a stable microenvironment and collecting reliable data over extended periods.^244^ This real-time monitoring also enables tracking liver functionality and cellular responses to drugs, facilitating the continuous assessment of key metabolic processes. For example, oxygen sensors are critical for managing zonation and for elucidating how different regions of the liver respond to drug exposure over time.^115^ Another example is the in situ biosensing of liver biomarkers such as albumin and GST-α, which is highly valuable.^250,251^ However, integrating these sensors into LoC systems presents several barriers; first, they must be carefully embedded to avoid disrupting microfluidic flow or compromising cell viability. Second, as more parameters are required to be monitored, the integration of multiple sensors into a compact chip becomes increasingly complex, requiring precise calibration and design to avoid interference between the sensors and biological elements

#### 5.2.4 Applications

Many LoC models have demonstrated significant promise in the detection of drug-induced liver injury (DILI),^1^ a critical concern in drug safety evaluations. s. For instance, some researchers showed that their LoC models have successfully predicted the hepatotoxicity of acetaminophen and troglitazone,^144,201^ whereas traditional models often fail. Additionally, in methotrexate (MTX)-treated chips, steatosis, an early indicator of metabolic liver disease, was observed, along with the activation of hepatic stellate cells (HSCs), leading to liver fibrosis.^252,253^ However, several challenges remain to be overcome; the limited number of cells per chip, makes downstream molecular analyses, such as proteomics or metabolomics, difficult to perform and often requires pooling cells from multiple chips, increasing costs, decreasing high-throughput capacities and constraining the ability of conducting comprehensive molecular and toxicological assessments^254^

Although LoC systems are promising for toxicology, their application in comprehensive drug metabolism studies, particularly ADMET and PK, faces several obstacles. The major one is metabolite profiling in long-term studies, where the accumulation of metabolites can affect drug behavior and complicate the accurate modeling of clearance rates. Advances in microfluidic designs have enabled better nutrient and waste exchange, as demonstrated when studying drugs such as diclofenac, where continuous perfusion prevented metabolite buildup, supporting a more reliable long-term profiling.^255^ Another key issue is replicating liver zonation, where differences in enzyme activity and oxygen gradients across liver zones are critical for drug metabolism, and toxicity studies. Drugs which undergo multiple metabolism phases, strongly depend on zonal representation to accurately reflect metabolic rates and drug clearance in different liver zones.^152,256^ Remarkably, some advanced LoC models reported in the previous sections have enabled a better simulation of this aspect. Notably, integrating LoC with other organ models, such as gut and heart chips, has enabled more comprehensive ADMET and PK studies.^255,257–259^ For example, a liver-skin model has better replicated first-pass metabolism, a crucial step affecting bioavailability and overall drug efficacy, which further underscores the potential of liver-based multiorgan systems for more accurate drug metabolism studies.^255,260^

#### 5.2.5 Culturing Issues

Limitations which affect long-term cell culturing ability and cell source reliability remain significant. PHH, though physiologically relevant, lose key functions such as CYP450 enzyme activity over time, which limits their use in long-term studies.^101^ Sourcing of these cells is also challenging owing to donor variability and limited availability. iPSCs offer a renewable alternative; however, they face reprogramming challenges that may result in incomplete differentiation into mature hepatocyte-like cells with low enzyme activity.^261^ Despite these issues, future advancements in iPSCs systems hold significant promise for personalized medicine by enabling researchers to generate patient-specific cells for drug testing and disease modeling. Culture medium selection is another important factor for co- to quad-culture LoC models, and those integrated with other organs. Currently, no universal CCM exists; instead, mixtures or supplemented standard media are used, which may reduce key component concentrations, making them unsuitable for sensitive PHH and iPSCs^1^

Bioprinting offers a noteworthy approach that enables high resolution, i.e. precise spatial arrangement of cells, including both iPSC-derived cells and PHH. Early bioprinted liver models have already shown improved cell organization and function, enhancing their potential for long-term studies. However, challenges related to scalability and consistency across printed tissues need to be considered.^192,244^

#### 5.2.6 Liver Organoid-Chips

Liver organoids, especially when incorporated into LoC systems, provide enhanced physiological relevance due to the inclusion of microfluidic control, perfusion, and shear stress. Yet, for the same reasons previously mentioned, cell culture remains one of the primary difficulties in constructing these models. iPSC-derived organoids exhibit an immature phenotype characterized by low CYP activity and reduced albumin production^262^

The second issue is related to the selection of the scaffold material which should provide both the structural support and biochemical cues necessary for proper organoid formation. Hydrogels, such as Matrigel, are commonly used to construct a 3D microenvironment that mimics the ECM of the liver. Although Matrigel effectively supports hepatocyte function, it has several drawbacks, including batch-to-batch variability and the presence of xenogeneic components derived from animal sources, which can affect reproducibility and limit its use in standardized liver organoid research.^190^ To resolve, synthetic hydrogels, such as hybrid PEG-based materials, are being explored as alternatives because they are free from animal-derived components, offering more consistent and reproducible environments.^263^ They also allow for the tuning of the mechanical properties and biochemical signals of the scaffold, ultimately leading to more physiologically relevant models. Additionally, hollow fiber membrane-based materials coated with recombinant human laminin have demonstrated improved hepatocyte maturation, enhancing critical liver functions, such as drug metabolism and transporter activity.^264^ These efforts represent an important step toward more standardized liver organoids; nonetheless, visualizing and analyzing the cell morphology, position, and function of these large 3D multicellular structures, especially over time and under dynamic flow, poses a significant difficulty.^190^ Continuous advancement of microscopic techniques and AI technology will enable improved organoid computer vision (OCV), which will mitigate this limitation.^265,266^

#### 5.2.7 Imaging Techniques

another vital aspect of LoC monitoring. For instance, high-resolution fluorescence microscopy facilitates real-time tracking of hepatotoxic effects such as lipid droplet formation in DILI.^197^ Despite such crucial roles, the current fluorescence microscopy methods face several issues related to LoC systems such as limited light penetration, challenging visualization of multicellular structures, and compromised cell viability due to continuous flow, light exposure and thermal effects. However, advanced techniques such as multiphoton microscopy (MPM) and light-sheet fluorescence microscopy (LSFM) permit deeper tissue penetration and high 3Dresolution with minimal photodamage; MPM uses near-infrared pulsed, whereas LSFM uses multiangle illumination. Additionally, label-free techniques such as hyperspectral stimulated Raman scattering (SRS) microscopy provide high-specificity chemical detection, offering new prospects for studying drug distribution and analyzing cellular heterogeneity in LoC models^267^

#### 5.2.8 Data Analysis

The ability to draw deep inferences from extensive data sets generated during long-term studies is increasingly important. Continuous monitoring produces enormous amounts of data, particularly in multicellular and complex LoCs, thus requiring advanced tools for analysis and interpretation such as AI-driven methods, specifically machine learning (ML) and deep learning (DL) algorithms.^268,269^ This emerging technology could greatly enhance the prediction of drug responses and liver functionality.^270,271^ However, as LoC systems advance, more sophisticated and accessible data-analysis tools are needed

#### 5.2.9 Clinical Validation

Besides, there are obstacles in applying the results of LoC models to clinical settings, including the requirement for validation against well-established in vivo models and clinical studies before general acceptance.^6^ Straight translation to clinical settings is limited by differences in liver physiology between humans and animals, and it is challenging to evaluate and validate results using available data because of their complexity.^101^ Therefore, LoC models, either academic or commercial, need to undergo comprehensive validation in order to receive regulatory permission before being used for safety assessment and medication development^272^

### 5.3 Toward Future LoC Models: Strategies and Recommendations

In the previous section, we described the challenges faced by different models of LoC, which are also commonly encountered in OoC, and outlined various efforts made by research institutions, companies, and consortiums to overcome them. In this section, we will further address these limiting barriers and propose concrete, actionable strategies, along with providing our recommendations on where efforts should be concentrated and which pathways are the most promising toward advanced LoC models in the future.

#### 5.3.1 Strategic Categorization and Standardization Pathways

The most critical obstacle facing standardization efforts is the diversity of LoC models. However, rather than following a one-size-fits-all approach, i.e., developing a single reference chip and forcing other models to conform, a categorization strategy of LoC models based on the intended applications (e.g., DILI screening, metabolism profiling, and tumor models) should be applied, followed by the establishment of performance metrics (e.g., CYP450 activity, albumin/urea production, and response kinetics) tailored to each category. Additionally, consensus guidelines on safety assurance, qualification pathways, minimum standards, and benchmark data toward ensuring reliability need to be established by current and future international consortia. Such categorization aligns directly with emerging regulatory programs like FDA’s MPS qualification and EMA’s NAM frameworks, where model credibility is evaluated relative to a defined context of use

#### 5.3.2 PDMS Optimization and Mass Production

As PDMS remains the dominant material in both academic and commercial models due to its critical advantages, it is not expected to be substituted. Thus, rather than prioritizing the development of novel alternative polymers, efforts should focus on mitigating its inherent disadvantages. This can be achieved through effective surface modification—to minimize small molecule absorption without significantly compromising O_2_ permeability—and by scaling up its fabrication using 3D printing or injection molding. The recent promising results obtained by some researchers in this area^275^ should be valued by OoC companies toward the industrialization of PDMS chip fabrication, which will help reduce variability in material properties and improve the overall reproducibility of their platforms

#### 5.3.3 Efficient Chip Sealing

To solve the issue of chip sealing, lock-and-play systems that ensure reversible, easy clamping using gripper levers emerge as a highly recommended option, as they offer a simple, single-step, and practical solution that effectively prevents leaks, deformation of the flexible microfluidic channels, and fluctuation of the flow regime.^276^ Notably, some researchers^147,177^ and manufacturers (e.g., CN-Bio) use open-well format systems, which allow simple implantation and easy end point analysis

#### 5.3.4 Multiplexed Nanosensing

To address the LoC monitoring issues, short-term strategies should rely on using robust off-chip sensors (optical O_2_/pH probes, pressure/flow units, and screen-printed electrochemical sensors) connected via minimal-dead-volume tubing for multiparametric readouts without any chip redesign. However, long-term efforts must focus on developing and integrating on-chip multiplexed nanosensors, such as graphene transistor arrays and plasmonic nanosensors, to achieve sensitive, simultaneous and near-real-time monitoring of O_2_, pH, metabolites, and liver-specific biomarkers, thereby providing a comprehensive model characterization essential for ML training and validation^268^

#### 5.3.5 Adaptive Designing and Monitoring

Concurrently, AI-driven tools can significantly contribute to the optimization of chip design, determination of perfusion parameters (i.e., optimal recirculating vs single-pass ratios and timing, flow rates, declogging, etc.), minimizing trial-and-error in experiments. Furthermore, future AI-powered automation will permit the dynamic regulation of these parameters, enabling prolonged cultures and improved throughput, thereby creating sophisticated OoC models

#### 5.3.6 Image Digitization

Time-lapse imaging without disrupting the LoC culture environment is also very important and can be achieved by microscopes featuring a stage-top incubator for short experiments or using microscope-based incubation systems, which ensure monitoring over several days.^277^ However, microscope manufacturers should consider chip connection with their perfusion system. To unlock the full potential of LoC technology, the obtained images should be used for training ML models to transform them from qualitative observations into quantitative data for fast, automated, and accurate interpretation. Up to date, a few studies have combined OoCs and DL,^195,278^ and mostly focused on image quality classification,^279^ video resolution enhancement,^280^ cell morphology for drug response quantification,^281^ OoC model qualification,^282^ cell trajectory tracking for determining cell–cell interactions^283^ and for drug efficacy evaluation.^284^ The algorithms used for DL trainings include recurrent neural networks (RNN),^279^ convolutional neural networks (CNNs),^280–282^ combined CNN-RNN,^284^ and generative adversarial networks (GANs).^283^ Notably, CNNs appear to be the most promising in addressing many of OoC challenges.^195^ We recommend using these approaches or developing similar ones specifically for LoC platforms in order to enhance hepatoxicity prediction, assess various drugs or immunotherapies, and ultimately accelerate the process of LoC model optimization and validation

#### 5.3.7 Scalable Long-Term Culturing

To mitigate the activity loss of PHH, several strategies have been identified, such as zonation-like oxygen gradients, ECM-matched hydrogels, and balanced coculture with NPCs, which have demonstrated efficacy in enhancing PHH stability. Furthermore, these sensitive cells should experience very low shear stress, like in the human liver. Therefore, the previously mentioned flow rate optimization and adjustment will significantly help maintain their function. LoC models that use large culture chambers (e.g., commercial CN-Bio, Dynamic42, or perfused well plates used by some researchers^147,177,194^ ) can produce large quantities of functional hepatocytes, which not only increases the cell number but also enhances the culture consistency and enables large sample volumes for multiomic analysis. The multichip plate configuration (e.g., academic^147,177^ and commercial CN-Bio, PREDICT96, MIMETAS) also resolves the low throughput capacity faced by sinusoid-like LoC models

Long-term culturing issues can also be addressed by bioprinting technology, whose future advances will resolve the current limitations and enable its efficient integration with OoC technology. For instance, robotic handling of microfluidic chips during the printing process will reduce manual intervention, enhancing consistency and scalability, as proven by Fritschen et al.^285^ Additionally, AI-optimized bioink formulations will ensure both mechanical and functional performance. Reproducible complex 3D constructs with high resolution can be obtained using laser-assisted bioprinting without reducing cell viability. Emergent 4D bioprinting is also very promising, since it promotes dynamic structural deformations such as the generation of vascular networks over time and the programming of drug release, which holds strong potential to transform LoC models for disease modeling (e.g., cancer) and drug testing.

#### 5.3.8 AI-Driven Clinical Validation

To overcome the dual challenges of data analysis and clinical validation of LoC models, DL should be highly integrated, and interdisciplinary collaboration among experts in bioengineering, computer science, clinical research, and pharmaceutical sciences should be fostered. First, OoC stakeholders are encouraged to obtain, organize, and share correct, high-quality and large experimental data, essential for effective DL training. Future developments are expected to yield automated data annotation systems and to benefit from self-supervised learning advancements that may eventually eliminate the need for labeling. However, until then, all obtained data should be well-labeled. Second, we recommend a pathway centered on deploying open-source digital twins (DTs) like DigiLoCS^286^ and layering them with AI-powered tools, in which MegaTox-like collections of valuable data sets and ML models can be very helpful. These AI-enhanced DTs must be systematically benchmarked against high-quality human clinical data. The development of an integrated toolkit that combines multimodal data from in vitro LoCs, DTs, and clinical sources will leverage the synergy of LoC technology, mechanistic modeling, and DL. When combined with a closed-loop approach for continuous refinement, this strategy represents one of the most promising pathways to improve the predictivity of disease progression and drug response, supporting the creation of clinically relevant LoC systems that can reliably reduce animal testing

In conclusion, significant efforts from both commercial and academic sectors are being undertaken to advance LoC models and to overcome their challenges. While there are promising solutions related to key features such as microfluidic materials, embedded multiplexed nanosensors, and AI-powered data analysis, LoC platforms still require considerable effort to demonstrate their accuracy in replicating human biology and predicting drug efficacy or toxicity. The development of clinically relevant, ready-to-use tools also involve substantial investment, as emphasized by recent NIH funding for prominent translational centers in partnership with the FDA.^10^

However, with ongoing collaboration, LoC systems are positioned to make significant contributions to minimizing animal testing, assessment costs, and ethical issues. As this technology continues to evolve, it is expected to deliver more widely adopted tools for healthcare and pharmaceutical applications.

## 6 Conclusions

The advancement of LoC technology marks a significant breakthrough in biomedical research, offering a remarkable ability to model human liver functions compared to traditional methods such as animal models and 2D cultures. Many LoC academic platforms and commercial products of leading companies such as Emulate, CN-Bio, Draper, and MIMETAS have been developed. They incorporate key features such as 3D multicellular architectures, vascular flow, and ECM, ensures the dynamic coculture and intertissue communication, making LoC models very useful tools for studying drug metabolism, toxicity, liver diseases, and personalized medicine.

These systems have successfully modeled both acute and chronic liver conditions such as fibrosis and cirrhosis, which are difficult to study using conventional methods. Moreover, the ability to integrate them with other organ models has expanded their utility beyond liver-specific studies. Multi-OoC models, such as CN-Bio’s liver coupled with gut or lung, provide insights into the systemic effects of drugs and diseases, allowing researchers to predict toxicity, efficacy, and pharmacokinetics in a human-relevant manner. Similarly, platforms such as TissUse’s HUMIMIC chips and Hesperos’ heart-liver and multiorgan models contribute to a deeper understanding of organ interactions, further enhancing their range of applications.

Furthermore, the regulatory landscape is also shifting. Notably, FDA’s ISTAND Pilot Program recently accepted the first organ-on-a-chip submission designed to predict human drug-induced liver injury (DILI), marking a critical step toward formal qualification of LoC platforms as drug development tools.^287^

Nevertheless, many challenges still persist. Challenges such as selecting the optimum microfluidic material, level of model complexity, hepatic cell source, standardization, and validation must be addressed. Much progress has been made in this regard. Multiplexing and integration of automated systems can enhance the reproducibility of LoC platforms and promote their standardization. These features present in commercial Draper’s PREDICT-96 and PhysioMimix allow for high-throughput testing, provide significant time and cost savings, and offer scalable solutions for healthcare institutes and companies to conduct preclinical testing with greater efficiency. Future developments will focus on making these models fully standardized, monitored, automated, mass produced, user-friendly, and most importantly, clinically validated. As LoC models continue to evolve, they will play an increasingly important role in reducing, refining, or even replacing the use of animal models according to the “3Rs”—an objective highly encouraged by ethical committees. However, achieving this goal requires close collaboration between academia, industry, funding institutions, and regulatory bodies.

## List of Abbreviations

3Rs: Replacement, Reduction, Refinement (ethical principles for animal testing)
ADH: Alcohol Dehydrogenase
ADMET: Adsorption, Distribution, Metabolism, Excretion, and Toxicity
AI: Artificial Intelligence
ALB: Albumin
ALD: Alcoholic Liver Disease
ALF: Acute Liver Failure
cccDNA: Covalently Closed Circular DNA
CCA: Cholangiocarcinoma
CNNs: Convolutional Neural Networks
COC: Cyclic Olefin Copolymer
CPBT: Centre for Animal-Free Biomedical Translation
CV: Central Vein
CYP or CYP450: Cytochrome P450
DAAs: Direct-Acting Antivirals
DAMPs: Damage-Associated Molecular Patterns
dECM: Decellularized Extracellular Matrix
DEP: Dielectrophoresis
DILI: Drug-Induced Liver Injury
DL: Deep Learning
DLP: Digital Light Processing
ECM: Extracellular Matrix
ESTEM: Cellular Therapy and Stem Cell Production Application and Research Center
FDA: U.S. Food and Drug Administration
FEP: Fluorinated Ethylene Propylene
GANs: Generative Adversarial Networks
GelMA: Gelatin Methacryloyl
GST-alpha: Glutathione S-Transferase Alpha
HA: Hepatic Artery
HBV: Hepatitis B Virus
HCC: Hepatocellular Carcinoma
HCs: Hepatocytes
HCV: Hepatitis C Virus
HGF: Hepatocyte Growth Factor
hiHep: Human-Induced Hepatocytes
hiPSC or hiPSCs: Human-Induced Pluripotent Stem Cell(s)
HSCs: Hepatic Stellate Cells
HUMIMIC: Human-on-a-Microfluidic-Chip
IGF-1: Insulin-like Growth Factor 1
IIDMP: Integrated Insert on a Dynamic Microfluidic Platform
KCs: Kupffer Cells
LADY: Liver Acinus Dynamic
LDL: Low-Density Lipoprotein
LLC or LLCs: Liver Lobule Chip(s)
LNPs: Lipid Nanoparticles
LoC: Liver-on-a-Chip
LPS: Lipopolysaccharide
LSECs: Liver Sinusoidal Endothelial Cells
mHAC: Microfluidic Hepatic Acinus Chip
MAFLD or MASLD: Metabolic Dysfunction-Associated Steatotic Liver Disease
ML: Machine Learning
MPS: Microphysiological Systems
NAFLD: Non-Alcoholic Fatty Liver Disease
NASH: Non-Alcoholic Steatohepatitis
NPCs: Non-Parenchymal Cells
OoC: Organ-on-a-Chip
PAMPs: Pathogen-Associated Molecular Patterns
PCL: Polycaprolactone
PDMS: Polydimethylsiloxane
PECAM-1: Platelet Endothelial Cell Adhesion Molecule-1
PHH or PHHs: Primary Human Hepatocyte(s)
PK: Pharmacokinetic
PMMA: Poly-(Methyl Methacrylate)
PTFE: Polytetrafluoroethylene
PV: Portal Vein
R&D: Research and Development
RNNs: Recurrent Neural Networks
SPION: Superparamagnetic Iron Oxide Nanoparticles
TNF-α: Tumor Necrosis Factor Alpha
TVLOC: Tri-Vascular Liver-on-a-Chip
UGT: UDP-Glucuronosyltransferase
VEGF: Vascular Endothelial Growth Factor
VLSLL: Very Large Scale Liver Lobule
